# Selective synthesis of α-organylthio esters and α-organylthio ketones from β-keto esters and sodium *S*-organyl sulfurothioates under basic conditions

**DOI:** 10.3762/bjoc.17.24

**Published:** 2021-01-26

**Authors:** Jean C Kazmierczak, Roberta Cargnelutti, Thiago Barcellos, Claudio C Silveira, Ricardo F Schumacher

**Affiliations:** 1 Departamento de Química, CCNE, Universidade Federal de Santa Maria–UFSM, Santa Maria, 97105-900, RS, Brazil; 2 Instituto de Biotecnologia Universidade de Caxias do Sul–UCS, Caxias do Sul, RS, Brazil

**Keywords:** α-alkylthio esters, α-alkylthio ketones, Bunte salts, C–C bond cleavage, β-keto esters

## Abstract

We described herein a selective method to prepare α-organylthio esters and α-organylthio ketones by the reaction of β-keto esters with sodium *S*-benzyl sulfurothioate or sodium *S*-alkyl sulfurothioate (Bunte salts) under basic conditions in toluene as the solvent at 100 °C. When 4 equivalents of a base were used, a series of differently substituted α-thio esters were obtained with up to 90% yield. On the other hand, employing 2 equivalents of a base, α-thio ketones were achieved after 18 h under air. Furthermore, after a shorter reaction time, the isolation of keto–enol tautomers was possible, revealing them as significant intermediates for the mechanism elucidation.

## Introduction

During the last ten years, sodium *S*-organyl sulfurothioates, also known as Bunte salts, were rediscovered by many researchers as stable, nonhygroscopic, and moisture-resistant thiolating agents. Therefore, they have been actively studied as precursors for the preparation of diverse sulfur-containing compounds [1]. These recent findings include their use in direct sulfenylation reactions of electron-rich *N*-heterocycles [2–4], decarboxylative cross-coupling reactions with propiolic acid derivatives [5], Michael addition reaction [6], cross-couplings catalyzed by Pd [7] and Cu salts [8–9], the preparation of symmetrical and nonsymmetrical disulfides [10–11], and the synthesis of β-acetamido sulfides by the acetamidosulfenylation of alkenes [12], among others [13–15].

Sulfur-containing compounds are important intermediates in organic synthesis, being able to act as an electrophile or nucleophile in many organic transformations [16–18]. Still, many of them are pharmacologically active as antibiotic, analgesic, anti-inflammatory, antidepressant, and antidiabetic agents [19–22].

**Figure 1:**
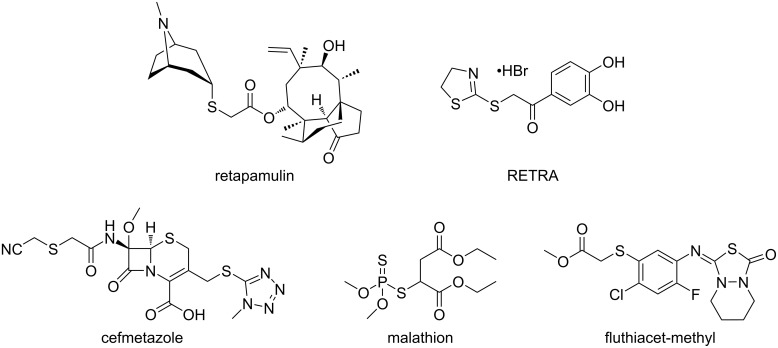
Drugs and agrochemicals containing the α-thiocarbonyl core as a structural motif.

In this regard, special attention can be given to α-thiocarbonyl compounds, which have appeared as synthons in many organic transformations [23–31] and as a core present in several worldwide-consumed drugs and agrochemicals [32–38]. Figure 1 shows examples of these pharmacologically active compounds, such as the antibiotics retapamulin and cefmetazole [32–35], the anticancer agent RETRA [36], and agrochemicals with pesticidal (malathion) and herbicidal applications (fluthiacet-methyl) [37–38].

**Scheme 1:**
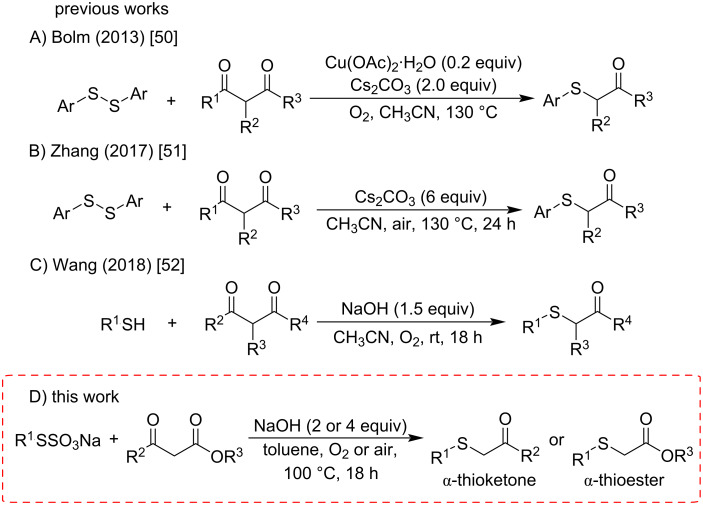
Methods for the synthesis of α-thiocarbonyl compounds by C**–**C bond cleavage of 1,3-dicarbonyl compounds.

In this sense, the development of efficient methodologies for the synthesis of sulfur-containing carbonyl compounds employing cheap, stable, nontoxic, easy-to-prepare, and easy-to-handle starting materials is crucial in contemporary organic synthesis and medicinal chemistry. In the last years, different methods have been developed to prepare these classes of molecules, which includes the reaction of alkynes [39–43] as well as α-halogenated [44–45] and α-diazo carbonyl compounds [46–49] with thiols, diorganyl disulfides, and related compounds. Some of them are catalyzed by expensive transition metals, such as gold, iridium, palladium, and titanium. More recently, the selective formation of C–S bonds using 1,3-dicarbonyl compounds, followed by a C–C bond cleavage has emerged as a versatile and less expensive protocol to prepare α-thiocarbonyl compounds. In this way, Bolm and collaborators elaborated a copper(II) acetate-catalyzed reaction of β-dicarbonyl compounds with diaryl disulfides (Scheme 1A) [50]. In 2017, Zou and co-workers described the preparation of α-thiocarbonyl compounds through a reaction of 1,3-dicarbonyl substrates with diaryl disulfides promoted by Cs_2_CO_3_ at 130 °C (Scheme 1B) [51]. A year later, Wang and co-workers demonstrated that β-dicarbonyl compounds could react with thiols at room temperature under an oxygen atmosphere in the presence of a base. This work also demonstrated an easy access to α-sulfenylated amides (Scheme 1C) [52].

Inspired by those elegant pioneering studies, we explored the reaction of 1,3-dicarbonyl compounds with Bunte salts mediated by a base (Scheme 1D). The choice of NaOH as the base and its concentration were crucial to the selective synthesis of α-thio esters or α-thio ketones starting from β-keto esters. It is worthwhile to note herein that although some excellent synthetic protocols have already been developed, there is a gap to be filled in the preparation of α-benzyl thiocarbonyl and α-alkyl thiocarbonyl compounds not fully explored by these recent methods. Thus, the main features of the current strategy include the use of Bunte salts, avoiding air-sensitive and foul-smelling starting materials [53–54] to react with β-keto esters under NaOH-mediation to selectively produce α-thio esters or α-thio ketones as target products. Additionally, no prefunctionalization of the carbonyl compounds was required.

## Results and Discussion

We started the investigation of the optimal conditions by varying the amount of the reagents and selecting the ideal base and solvent. For that, we chose ethyl acetoacetate (**1a**) and sodium *S*-benzyl sulfurothioate (**2a**) as the standard starting materials to establish the best reaction conditions under an air atmosphere (Table 1).

**Table 1:** Optimization of the reaction conditions.^a^

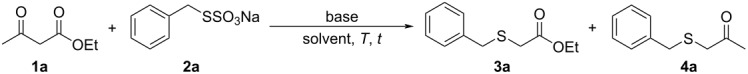						

entry	**1a**, mmol	**2a**, mmol	base, equiv	solvent	yield of **3a**, %	yield of **4a**, %

1	1.0	0.5	NaOH (2)	toluene	20	44
2	1.0	0.5	KOH (2)	toluene	20	27
3	1.0	0.5	K_3_PO_4_ (2)	toluene	32	21
4	1.0	0.5	K_2_CO_3_ (2)	toluene	–	31
5	1.0	0.5	Et_3_N (2)	toluene	4	–
6	1.0	0.5	DBU (2)	toluene	–	–
7	1.0	0.5	Cs_2_CO_3_ (2)	toluene	23	–
8	1.0	0.5	Na_2_CO_3_ (2)	toluene	15	–
9	1.0	0.5	KF (2)	toluene	15	–
10	1.0	0.5	NaOH (1)	toluene	18	21
11	1.0	0.5	NaOH (4)	toluene	48	traces
12	1.0	0.5	NaOH (4)	MeCN	34^b^	20
13	1.0	0.5	NaOH (4)	dioxane	20	–
14	1.0	0.5	NaOH (4)	DMF	–	–
15	1.0	0.5	NaOH (4)	DMSO	–	–
16	1.0	0.5	NaOH (4)	THF	36^b^	–
17	1.0	0.5	NaOH (4)	DCM	20^b^	–
18	1.0	0.5	NaOH (4)	EtOH	traces	traces
19	1.0	0.5	NaOH (4)	acetone	30	–
20	0.5	1.0	NaOH (4)	toluene	50	23
21	0.5	1.5	NaOH (4)	toluene	50	40
22	0.5	1.0	NaOH (4)	toluene^c^	86	–
23	0.5	1.0	NaOH (2)	toluene	11	68
24	0.5	1.0	NaOH (2)	toluene^c^	17	42
25	0.5	1.0	NaOH (2)	toluene^d^	47	37
26	0.5	1.0	NaOH (4)	toluene^d^	55	34
27	0.5	1.0	–	toluene	–	–

^a^The reactions were conducted using ethyl acetoacetate (**1a**), sodium *S*-benzyl sulfurothioate (**2a**), base, and solvent (3 mL) at 100 °C under air for 18 h. ^b^Reaction conducted at reflux temperature. ^c^Reaction conducted under an O_2_ atmosphere. ^d^Reaction conducted under an N_2_ atmosphere.

In our preliminary experiment, a mixture of **1a** (1.0 mmol) and **2a** (0.5 mmol), using NaOH (2 equiv) as a base in toluene (3.0 mL), was stirred at 100 °C for 18 h. Under these reaction conditions, the products ethyl 2-(benzylthio)acetate (**3a**, 20% yield) and 1-(benzylthio)propan-2-one (**4a**, 44% yield) were obtained in a pure form by column chromatography (Table 1, entry 1). Interestingly, the formation of both an α-thio ester and an α-thio ketone, starting from ethyl acetoacetate and a sulfur source, was not reported by the previous literature shown in Scheme 1. As such, intrigued by this result and aiming to find selective methods to prepare both α-thio carbonyl compounds (**3a** and **4a**), we decided to verify the influence of different inorganic and organic bases on the reaction (Table 1, entries 2–9). During these experiments, neither increments on the reaction yield nor on the selectivity with respect to the obtained the carbonyl compounds were observed. Next, we decided to evaluate the amount of NaOH by decreasing the amount of base to 1 equiv as well as increasing it to 4 equiv (Table 1, entries 10 and 11). A close inspection showed that 4 equiv of NaOH increased the amount of ethyl 2-(benzylthio)acetate (**3a**) to 48% and only traces of the product **4a** were observed. This result demonstrated the first insight into the selective formation of the carbonyl compound **3a** based on the amount of base. With this result in hand and considering 4 equivalents of NaOH as ideal for the formation of **3a**, we then explored the influence of diverse solvents (Table 1, entries 12–19). It can be observed that none of the tested solvents affected the reaction positively. In general, it was observed that polar and protic solvents gave a lower yield when compared to nonpolar and aprotic ones. Keeping toluene as the best solvent, we turned our attention to the amount of **1a** and **2a** (Table 1, entries 20 and 21). When the mixture of **1a** (0.5 mmol) and **2a** (1.0 mmol) reacted in the presence of NaOH (4 equiv), the product **3a** was obtained in 50% yield, along with **4a** in a yield of 23% (Table 1, entry 20). When the amount of *S*-benzyl sulfurothioate (**2a**) was increased to 3 equiv, we found an almost complete consumption of **1a**, however, lowering the selectivity of the reaction (Table 1, entry 21). In light of the previous results by Bolm et al. [45] and Wang et al. [47], we turned to conduct the reaction under an oxygen atmosphere (Table 1, entry 22). To our delight, the product **3a** was obtained selectively in 86% yield. In this case, even GC–MS analysis of the crude sample did not show the presence of **4a**. With the best conditions to obtain ethyl 2-(benzylthio)acetate (**3a**) selectively and in a high yield in hand (Table 1, entry 22), we turned our attention to find an ideal protocol to synthesize **4a**. When 0.5 mmol of **1a** and 1 mmol of **2a** were used in the presence of 2 equivalents of NaOH under air, compound **4a** was obtained in 68% yield (Table 1, entry 23). In this case, 11% of **3a** was also isolated. We considered Table 1, entry 23 as the best conditions to produce 1-(benzylthio)propan-2-one (**4a**). Lastly, the oxygen or nitrogen atmosphere was prejudice to the formation of **4a**, and in the absence of base, no product was obtained (Table 1, entries 24–27). At a lower temperature or at a shorter reaction time, the consumption of the starting materials and the decarboxylation process were incomplete.

Having established the ideal reaction conditions, we first proceeded to examine the reaction of the different β-keto esters **1a**–**i** with the sodium *S*-organyl sulfurothioates **2a**–**j** to produce a series **3a**–**r** of α-organylthio esters (Table 2). Initially, we focused on exploring diverse sodium *S*-benzyl sulfurothioates bearing neutral, electron-donating, and electron-withdrawing substituents in the *ortho*-, *meta*-, and *para*-positions. All reactions occurred smoothly, and the expected α-thio esters were obtained in moderate to excellent yield. A close inspection showed that in general, benzyl groups bearing electron-withdrawing groups (i.e., in **2b**–**e**) gave a lower yield when compared to neutral (i.e., in **2a**) and the electron-donating substituents (i.e., in **2f**–**i**, Table 2, entries 1–9). These results also suggest that the reaction is not sensitive to steric effects since no consistent results were obtained comparing the *ortho*- and *para*-substituted benzyl groups (see entries 2 vs 3 and 7 vs 8 in Table 2). Beyond benzyl groups, the sodium *S*-butyl sulfurothioate **2j** was also found to be a suitable reagent for this methodology, providing the corresponding ethyl 2-(butylthio)acetate (**3j**) in 40% yield (Table 2, entry 10).

**Table 2:** Substrate scope for the synthesis of the α-organylthio esters **3**.^a^

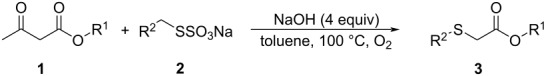				

entry	β-keto ester **1**	Bunte salt **2**	product **3**	yield, % (*t*, h)

1	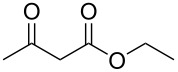 **1a**	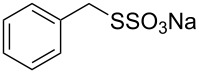 **2a**	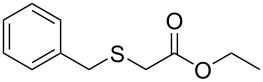 **3a**	86 (18)
2	**1a**	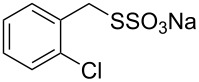 **2b**	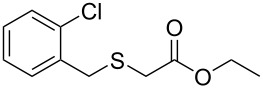 **3b**	62 (18)
3	**1a**	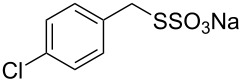 **2c**	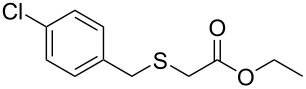 **3c**	78 (18)
4	**1a**	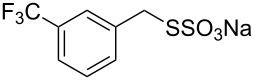 **2d**	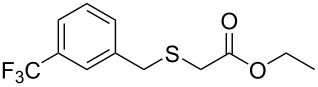 **3d**	60 (19)
5	**1a**	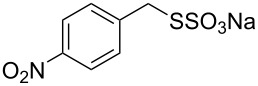 **2e**	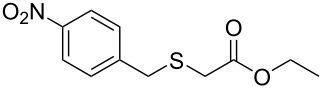 **3e**	45 (19)
6	**1a**	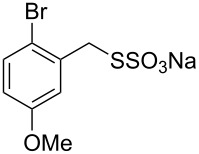 **2f**	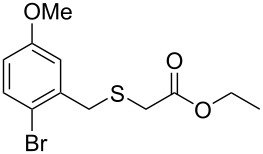 **3f**	68 (20)
7	**1a**	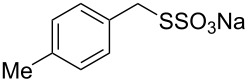 **2g**	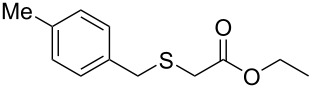 **3g**	75 (22)
8	**1a**	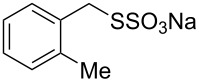 **2h**	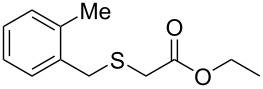 **3h**	90 (18)
9	**1a**	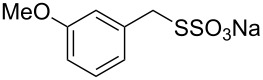 **2i**	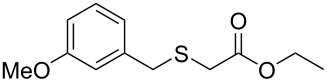 **3i**	74 (19)
10	**1a**	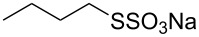 **2j**	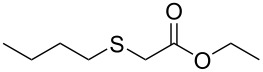 **3j**	40 (20)
11	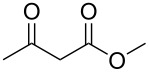 **1b**	**2a**	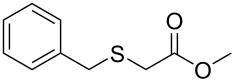 **3k**	35 (18)
12	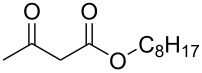 **1c**	**2a**	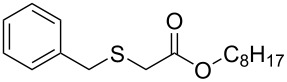 **3l**	44 (22)
13	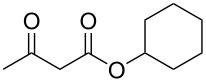 **1d**	**2a**	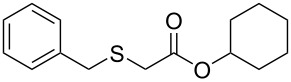 **3m**	42 (22)
14	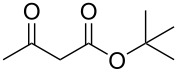 **1e**	**2a**	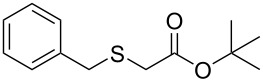 **3n**	70 (18)
15	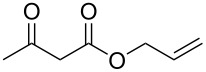 **1f**	**2a**	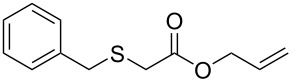 **3o**	20 (19)
16	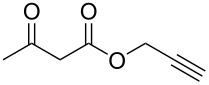 **1g**	**2a**	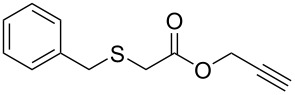 **3p**	–^b^ (18)
17	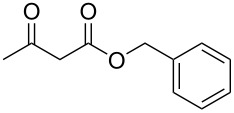 **1h**	**2a**	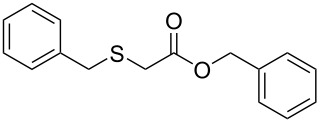 **3q**	44 (20)
18	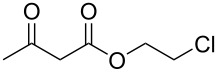 **1i**	**2a**	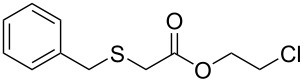 **3r**	traces (19)
19	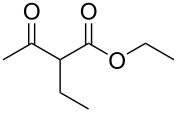 **1j**	**2a**	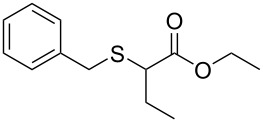 **3s**	– (18)

^a^The reactions were conducted using a β-keto ester **1** (0.5 mmol), a sodium *S*-organyl sulfurothioate **2** (1.0 mmol), NaOH (4 equiv), and toluene (3 mL) at 100 °C under an O_2_ atmosphere. ^b^The materials decomposed during the reaction.

We next surveyed a range of differently substituted β-keto esters in the reaction with sodium *S*-benzyl sulfurothioate (**2a**, Table 2, entries 11–18). This protocol enabled the synthesis of various α-benzylthio esters **3k**–**r** bearing alkyl, allyl, and benzyl groups directly bonded to the oxygen atom of the ester group. It can be noted that these products were obtained in moderate to good yields. When methyl, octyl, and cyclohexyl 3-oxobutanoates **1b**–**d** reacted with sodium *S*-benzyl sulfurothioate (**2a**), the expected products **3k**–**m** were obtained in only moderate yields (Table 2, entries 11–13). On the other hand, the reaction conducted using the tertiary-alkyl-substituted 3-oxobutanoate (**1e**) gave *tert*-butyl 2-(benzylthio)acetate (**3n**) in 70% yield (Table 2, entry 14). We also noted that the hybridization of the adjacent carbon atom bonded to the ester group had a direct effect on the formation of the product **3**. For instance, comparing entry 1 with entries 15 and 16 in Table 2, we observed a sudden decrease in the reaction yield, which, we believe, is related to the stability of the starting materials and the formed products in the reaction media. Furthermore, a benzyl group directly bonded to the ester **1h** was also employed under the standard reaction conditions, and after 20 h, benzyl 2-(benzylthio)acetate (**3q**) was isolated in 44% yield (Table 2, entry 17). When chloro-substituted ethyl acetoacetate **1i** was used to react with **2a**, only traces of the product **3r** were detected (Table 2, entry 18). Finally, limitations were observed also for ethyl 2-ethyl-3-oxobutanoate (**1j**), which failed to give the product **3s** (Table 2, entry 19).

Subsequently, we faced the challenge of forming the α-organylthio ketones **4** in a base-controlled selective reaction (Table 3). Under the standard reaction conditions (Table 1, entry 22), the products **4a**–**g** were obtained in moderate to good yield after chromatographic purification. When we performed the reactions with differently substituted sodium *S*-benzyl sulfurothioates, the products **4a**–**e** were obtained in yields ranging from 45% to 84% (Table 3, entries 1–5). We also tested methyl acetoacetate (**1b**) as a starting material to produce 1-(benzylthio)propan-2-one (**4a**). Interestingly, by this reaction, the product **4a** was produced in 80% yield. This suggests that methyl acetoacetate (**1b**) is also an appropriate substrate to produce the α-thio ketones **4**. Then, we turned our attention to the use of ethyl 4-chloro-3-oxobutanoate (**1k**) as a starting material to produce 1-(benzylthio)-3-chloropropan-2-one (**4f**). Unfortunately, after conducting this reaction, only trace amounts of **4f** were obtained, and decomposition of the carbonyl compounds was observed. Thereafter, we explored the potential of the methodology for the ethyl 3-oxo-3-arylpropanoates **1l** and **1m**. When substrate **1l**, containing an unsubstituted phenyl ring, was used, product **4g** was obtained in 50% yield after 20 h (Table 3, entry 8). On the other hand, the reaction conducted with halo-substituted substrate **1m** did not give the expected product, and the dicarbonylated starting material was recovered untouched. The reaction mixtures were chromatographed on silica gel, eluting with hexanes/EtOAc 99:1 to isolate the product **4** after combining the appropriate fractions. Minor fractions of the corresponding compound **3** were isolated in 6% to 17% yield.

**Table 3:** Substrate scope for the synthesis of the β-organylthio ketones **4**.^a^

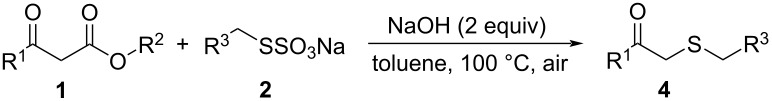				

entry	β-keto ester **1**	Bunte salt **2**	product **4**	yield, % (*t*, h)

1	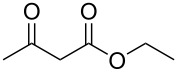 **1a**	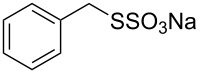 **2a**	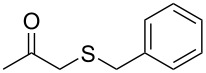 **4a**	68 (18)
2	**1a**	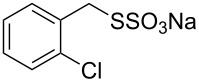 **2b**	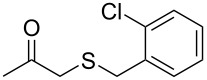 **4b**	84 (18)
3	**1a**	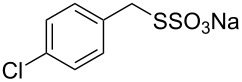 **2c**	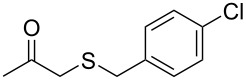 **4c**	45 (20)
4	**1a**	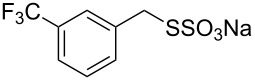 **2d**	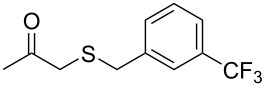 **4d**	70 (18)
5	**1a**	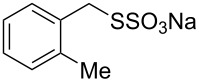 **2h**	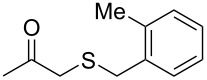 **4e**	70 (18)
6	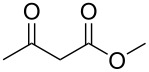 **1b**	**2a**	**4a**	80 (18)
7	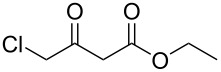 **1k**	**2a**	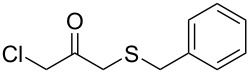 **4f**	traces (20)
8	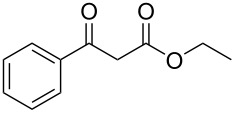 **1l**	**2a**	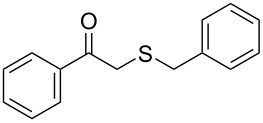 **4g**	50 (20)
9	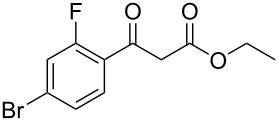 **1m**	**2a**	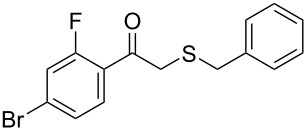 **4h**	– (20)
10	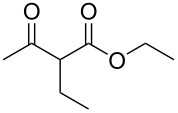 **1j**	**2a**	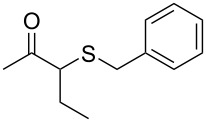 **4i**	– (20)

^a^The reactions were conducted using a β-keto ester **1** (0.5 mmol), a sodium *S*-organyl sulfurothioate **2** (1.0 mmol), NaOH (2 equiv), and toluene (3 mL) at 100 °C under air.

**Scheme 2:**
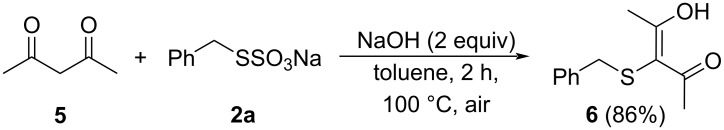
Formation of the enol **6** from acetylacetone (**5**).

In addition, we employed acetylacetone (**5**) as a reagent to obtain an α-thio ketone (Scheme 2). However, when compound **5** reacted with sodium *S*-benzyl sulfurothioate (**2a**), the enol product **6** was obtained in 86% yield after 2 h at 100 °C. In this case even a longer reaction time and a higher temperature were not effective to achieve the C–C bond cleavage.

**Scheme 3:**
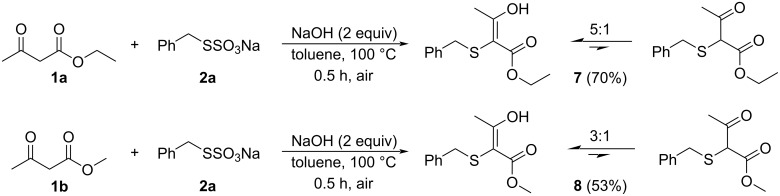
Formation of thio-substituted keto–enol tautomers **7** and **8**.

This interesting result also gave us the opportunity to study the formation of keto–enol tautomers, starting from the β-keto esters **1a** and **1b**. Then, conducting the reactions using 2 equiv of NaOH and toluene as the solvent at 100 °C for only 30 minutes, the products **7** and **8** were obtained in 70% and 53% yield, respectively (Scheme 3). The ratio between the keto and the enol tautomer is given based on the ^1^H NMR analysis. These results are consistent with the report by Tan and collaborators [55] and are evidence to help understand the reaction mechanism.

**Scheme 4:**
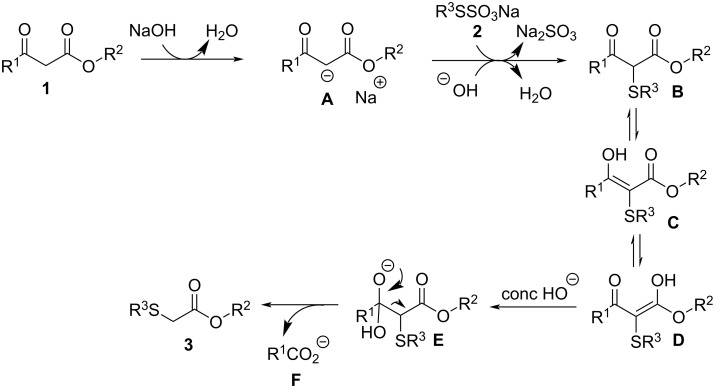
Proposed mechanism for the synthesis of **3**.

**Scheme 5:**
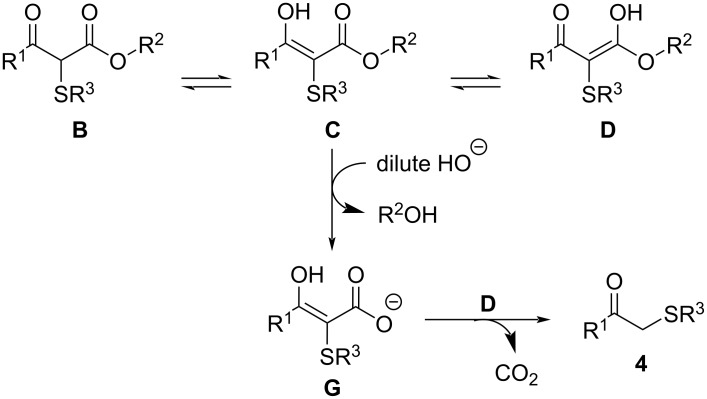
A tentative pathway for the synthesis of **4**.

Although detailed mechanisms for these reactions remain to be elucidated, the literature has clearly demonstrated that many transformations involving β-keto esters are dependent on the strength and concentration of the base [56–64]. Therefore, based on the above results and on the information from the literature, we propose the following mechanism for the synthesis of **3** (Scheme 4) [56–60] and **4** (Scheme 5) [61–64]: We believe that initially, an acidic hydrogen atom bonded to the methylene group between the two carbonyl groups in the β-keto ester **1** is removed by a HO^−^ anion to generate the stabilized carbanion intermediate **A**. The carbanion then attacks the most available sulfur atom of the sodium *S*-organyl sulfurothioate **2** to produce the keto−enol tautomers **B**, **C**, and **D**. Subsequently, when 4 equiv of NaOH (concentrated solution) are used, the most electron-deficient carbonyl group experiences a quick attack by the HO^−^ anion, generating the unstable tetrahedral intermediate ion **E**, the breakdown of which leads to a negatively charged 2-organylthioacetate stabilized by both a carbonyl group and the sulfur atom. Finally, a proton abstraction from the reaction medium produces the target product **3**. We showed that oxygen plays an important role in the selective formation of **3**, but the exact operation of O_2_ during this process remains unclear at this stage.

A tentative pathway for the formation of compound **4** is proposed in Scheme 5. It is well documented that with a dilute amount of base, ester hydrolysis followed by saponification takes place in β-keto esters, which is followed by CO_2_ displacement under heating [61–64]. We assume that in our case, when only 2 equiv of base are used, the keto–enol tautomer intermediates undergo a hydrolysis–decarboxylation process preferentially to a retro-Claisen reaction. This process forms the 3-oxocarboxylate ion **G**, which decarboxylates at a high temperature, providing the product **4**.

Finally, to validate the role of the Bunte salt as a sulfur source and to gain further insights into the mechanisms, several control experiments were performed, as can be seen in Supporting Information File 1.

## Conclusion

In conclusion, we developed simple, efficient, and selective methods for the synthesis of α-thio esters and α-thio ketones through NaOH-mediated C–S bond formation, followed by C–C bond cleavage. A broad range of β-keto esters and sodium *S*-organyl sulfurothioates were used as starting materials. Under the optimized reaction conditions, β-keto esters were shown to be multifaceted reagents suitable to produce α-thio esters or α-thio ketones, with the amount of base controlling the selective C–C bond cleavage. Sodium *S*-organyl sulfurothioates were satisfactorily used as a sulfur source that is free of unpleasant odor and not air-sensitive. The products were obtained in yields ranging from moderate to excellent. Keto–enol tautomers were isolated and identified as possible reaction intermediates. All obtained products were fully characterized by NMR and HRMS techniques. Although oxygen plays an important part in the selectivity, the exact role of O_2_ is currently not clear.

## Experimental

See Supporting Information File 1 for full experimental data of compounds **3**, **4**, and **6**–**8**.

## Supporting Information

File 1 Experimental procedures, characterization data, control experiments, and copies of the ^1^H, ^13^C, and ^19^F NMR spectra.
