# Supplementary material for: Selective synthesis of α-organylthio esters and α-organylthio ketones from β-keto esters and sodium S-organyl sulfurothioates under basic conditions
Source: Beilstein J Org Chem. 2021 Jan 26;17:234–44. doi: 10.3762/bjoc.17.24 (PMC7849251; doi:10.3762/bjoc.17.24)

BEILSTEIN JOURNAL OF ORGANIC CHEMISTRY

Supporting Information

for

Selective synthesis of α-organylthio esters and α-organylthio ketones from β-keto esters and sodium *S*-organyl sulfurothioates under basic conditions

Jean C. Kazmierczak, Roberta Cargnelutti, Thiago Barcellos, Claudio C. Silveira and Ricardo F. Schumacher

Beilstein J. Org. Chem. **2021,** *17,* 234–244. doi:10.3762/bjoc.17.24

Experimental procedures, characterization data, control experiments, and copies of the ^{1}H, ^{13}C, and ^{19}F NMR spectra

License and Terms: This is a supporting information file under the terms of the Creative Commons Attribution License (https://creativecommons.org/licenses/by/4.0). Please note that the reuse, redistribution and reproduction in particular requires that the author(s) and source are credited and that individual graphics may be subject to special legal provisions.
The license is subject to the *Beilstein Journal of Organic Chemistry* terms and conditions: (https://www.beilstein-journals.org/bjoc/terms)

Table of Contents

General Methods ………………………………………………………………........ S2
Typical procedure for the synthesis of α-thioesters **3** ……………………….…. S2
Typical procedure for the synthesis of α-thioketones **4** …………………….…. S7
Typical procedure for the synthesis of **6**, **7** and **8** ……………………….……. S8
Control experiments …………………………………………………….……... S9
References ……………………………………………………………….……... S12
Copies of ^{1}H, ^{13}C{1H} and ^{19}F NMR spectra ………………………….…… S13

General Methods

Commercial reagents were used without further purification. Bunte salts **2** were prepared based on literature procedures.[1] All reactions were monitored by thin layer chromatography (TLC) using silica gel plates (silica gel 60 F254 0.25 mm), and components were visualized by observation under UV light (254 and 365 nm) followed by spraying with acidic vanillin solution. Hydrogen nuclear magnetic resonance spectra (^{1}H NMR) were obtained on Bruker Avance III HD 400 MHz employing a direct broadband probe at 400 MHz. The spectra were recorded in $CDCl_3$ solutions. The chemical shifts are reported in ppm, referenced to tetramethylsilane (TMS) as the internal reference. Coupling constants (*J*) are reported in Hertz. Carbon-13 nuclear magnetic resonance spectra (^{13}C NMR) were obtained on Bruker Avance III HD 400 MHz employing a direct broadband probe at 100 MHz. The chemical shifts are reported in ppm, referenced to the solvent peak of $CDCl_3$ (δ 77.0 ppm). Fluor-19 nuclear magnetic resonance spectra (^{19}F NMR) were obtained on Bruker Avance III HD 400 MHz employing a direct broadband probe at 376 MHz. The chemical shifts are reported in ppm, referenced to 2-fluorobenzaldehyde (δ -122.4 ppm) as the external reference.[2] The high-resolution electrospray ionization mass spectrometry (ESI-QTOF) analysis were performed on a Bruker Daltonics micrOTOF-Q II instrument in positive mode. The samples were solubilized in HPLC-grade acetonitrile and injected into the APCI source by means of a syringe pump at a flow rate of 5.0 µL min-1. The follow instrument parameters were applied: capillary and cone voltages were set to +3500 V and -500 V, respectively, with a desolvation temperature of 180 ºC. For data acquisition and processing, Compass 1.3 for micrOTOF-Q II software (Bruker Daltonics, USA) was used. The data were collected in the m/z range of 50-1200 at the speed of two scans per second. Low-resolution mass spectra were obtained with a Shimadzu GC-MS-QP2010 mass spectrometer. Melting point (mp) values were measured in a Marte PFD III instrument with a 0.1 ºC precision.

Typical procedure for the synthesis of α-thioesters 3: To a reaction tube equipped with a stir bar containing a solution of β-keto ester **1** (0.5 mmol) in toluene (3.0 mL) were added sodium *S*-organyl sulfothioate **2** (1.0 mmol) and NaOH (2.0 mmol, 0.080 g). Oxygen was bubbled in the reaction mixture, which was stirred at 100 °C for 18-22 h. After being cooled to room temperature, the resulting mixture was quenched with

water (20 mL) and extracted with ethyl acetate (3 x 20 mL). The combined organic phases were washed with brine (20 mL), dried over anhydrous $MgSO_4$, filtered, and evaporated under reduced pressure. The residue was purified by column chromatography on silica gel with hexane/ethyl acetate (99:1) as eluent to give **3**.

Ethyl 2-(benzylthio)acetate (3a):[3] The product was isolated as a colorless oil. Yield: 0.09 g (86%). ^{1}H NMR ($CDCl_3$, 400 MHz): δ (ppm) 7.40–7.20 (m, 5H), 4.17 (q, J = 7.1 Hz, 2H), 3.83 (s, 2H), 3.06 (s, 2H), 1.28 (t, J = 7.1 Hz, 3H). ^{13}C{^{1}H} NMR ($CDCl_3$, 100 MHz): δ (ppm) 170.4, 137.3, 129.1, 128.5, 127.2, 61.2, 36.4, 32.4, 14.1. MS (EI, 70 eV; m/z (relative intensity)): 210 (24), 137 (10), 123 (97), 91 (100), 65 (20). HRMS: calcd for $C_{11}H_{14}O_2S$ (ESI-TOF, $[M + Na]^+$), 233.0607; found, 233.0607.

Ethyl 2-((2-chlorobenzyl)thio)acetate (3b): The product was isolated as a yellow oil. Yield: 0.076 g (62%). ^{1}H NMR ($CDCl_3$, 400 MHz): δ (ppm) 7.41-7.35 (m, 2H), 7.25-7.17 (m, 2H), 4.19 (q, J = 7.1 Hz, 2H), 3.96 (s, 2H), 3.14 (s, 2H), 1.29 (t, J = 7.1 Hz, 3H). ^{13}C{^{1}H} NMR ($CDCl_3$, 100 MHz): δ (ppm) 170.3, 135.1, 134.2, 131.0, 130.0, 128.7, 126.7, 61.4, 34.0, 32.8, 14.1. MS (EI, 70 eV; m/z (relative intensity)): 246 (13), 244 (33), 159 (33), 157 (86), 127 (34), 125 (100). HRMS: calcd for $C_{11}H_{13}ClO_2S$ (ESI-TOF, $[M+Na]^+$), 267.0217; found, 267.0209.

Ethyl 2-((4-chlorobenzyl)thio)acetate (3c):[4] The product was isolated as a light yellow oil. Yield: 0.094 g (78%). ^{1}H NMR ($CDCl_3$, 400 MHz): δ (ppm) 7.30-7.26 (m, J = 4H), 4.17 (q, J = 7.1 Hz, 2H), 3.79 (s, 2H), 3.06 (s, 2H), 1.28 (t, J = 7.1 Hz, 3H). ^{13}C{^{1}H} NMR ($CDCl_3$, 100 MHz): δ (ppm) 170.2, 135.8, 133.0, 130.5, 128.6, 61.3, 35.6, 32.2, 14.1. MS (EI, 70 eV; m/z (relative intensity)): 246 (6), 244 (17), 159 (24), 157 (66), 127 (32), 125 (100). HRMS: calcd for $C_{11}H_{13}ClO_2S$ (ESI-TOF, $[M+Na]^+$), 267.0217; found, 267.0213.

Ethyl 2-((3-(trifluoromethyl)benzyl)thio)acetate (3d): The product was isolated as a colorless oil. Yield: 0.082 g (60%). ^{1}H NMR ($CDCl_3$, 400 MHz): δ (ppm) 7.61(s, 1H), 7.53 (t, J = 7.8 Hz, 2H), 7.44 (t, J = 7.8 Hz, 1H), 4.18 (q, J = 7.1 Hz, 2H), 3.88 (s, 2H), 3.06 (s, 2H), 1.2 (t, J = 7.1 Hz, 3H). ^{13}C{^{1}H} NMR ($CDCl_3$, 100MHz): δ (ppm) 170.0, 138.4, 132.5, 130.9 (q, J = 32.3 Hz), 129.0, 125.8 (q, J = 3.8 Hz), 124.1 (q, J = 3.8 Hz),

124.0 (q, J = 272.4 Hz), 61.4, 35.9, 32.3, 14.1. ^{19}F NMR ($CDCl_3$, 376 MHz,): δ (ppm) -63,1. MS (EI, 70 eV; m/z (relative intensity)): 278 (36), 191 (70), 159 (100), 109 (36), 88 (90). HRMS: calcd for $C_{12}H_{13}F_3O_2S$ (ESI-TOF, $[M+Na]^+$), 301.0480; found 301.0475.

Ethyl 2-((4-nitrobenzyl)thio)acetate (3e): The product was isolated as a yellow oil. Yield: 0.056 g (45%). ^{1}H NMR ($CDCl_3$, 400 MHz): δ (ppm) 8.19 (d, J = 8.9 Hz, 2H), 7.53 (d, J = 8.9 Hz, 2H), 4.19 (q, J = 7.1 Hz, 2H), 3.92 (s, 2H), 3.06 (s, 2H), 1.29 (t, J = 7.1, 3H). ^{13}C{^{1}H} NMR ($CDCl_3$, 100 MHz): δ (ppm) 169.8, 147.2, 145.0, 129.9, 123.7, 61.4, 35.6, 32.2, 14.1. MS (EI, 70 eV; m/z (relative intensity)): 255 (39), 136 (27), 89 (30), 88 (100), 70 (30). HRMS: calcd for $C_{11}H_{13}NO_4S$ (ESI-TOF, $[M+Na]^+$), 278.0457; found 278.0457.

Ethyl 2-((2-bromo-5-methoxybenzyl)thio)acetate (3f): The product was isolated as a colorless oil. Yield: 0.109 g (68%). ^{1}H NMR ($CDCl_3$, 400 MHz): δ (ppm) 7.44 (d, J = 8.8 Hz, 1H), 6.98 (d, J = 3.1 Hz, 1H), 6.69 (dd, J = 8.8, 3.1 Hz, 1H), 4.19 (q, J = 7.1 Hz, 2H), 3.91 (s, 2H); 3.79 (s, 3H), 3.15 (s, 2H), 1.29 (t, J = 7.1 Hz, 3H). ^{13}C{^{1}H} NMR ($CDCl_3$, 100 MHz): δ (ppm) 170.3, 158.8, 137.5, 133.7, 116.7, 114.9, 114.7, 61.4, 55.5, 36.7, 32.7, 14.1. MS (EI, 70 eV; m/z (relative intensity)): 320 (39), 318 (37), 233 (100), 231 (99), 201 (67), 199 (69). HRMS: calcd for $C_{12}H_{15}BrO_3S$ (ESI-TOF, $[M+Na]^+$), 340.9817; found 340.9826.

Ethyl 2-((4-methylbenzyl)thio)acetate (3g):[4] The product was isolated as a yellow oil. Yield: 0.083 g (75%). ^{1}H NMR ($CDCl_3$, 400 MHz): δ (ppm) 7.21 (d, J = 7.8 Hz, 2H), 7.12 (d, J = 7.8 Hz, 2H), 4.17 (q, J = 7.1 Hz, 2H), 3.79 (s, 2H), 3.06 (s, 2H), 2.33 (s, 3H), 1.28 (t, J = 7.1 Hz, 3H). ^{13}C{^{1}H} NMR ($CDCl_3$, 100 MHz): δ (ppm) 170.4, 136.9, 134.1, 129.2, 129.0, 61.2, 36.0, 32.3, 21.0, 14.1. MS (EI, 70 eV; m/z (relative intensity)): 225 (3), 224 (16), 137 (72), 105 (100), 77 (13). HRMS: calcd for $C_{12}H_{16}O_2S$ (ESI-TOF, $[M+Na]^+$), 247.0763; found 247.0775.

Ethyl 2-((2-methylbenzyl)thio)acetate (3h):[5] The product was isolated as a yellow oil. Yield: 0.103 g (90%). ^{1}H NMR ($CDCl_3$, 400 MHz): δ (ppm) 7.24-7.21 (m, 1H), 7.18-7.11 (m, 3H), 4.19 (q, J = 7.1 Hz, 2H), 3.85 (s, 2H), 3.11 (s, 2H), 2.40 (s, 3H), 1.30 (t,

J = 7.1 Hz, 3H). $^{13}C\{^1H\}$ NMR ($CDCl_3$, 100 MHz): *δ* (ppm) 170.5, 136.9, 134.8, 130.7, 130.0, 127.5, 125.8, 61.3, 34.5, 32.7, 19.0,14.1. MS (EI, 70 eV; m/z (relative intensity)): 225 (3), 224 (20), 137 (45), 105 (100), 104 (52). HRMS: calcd for $C_{12}H_{16}O_2S$ (ESI-TOF, $[M+Na]^+$), 247.0763; found 247.0771.

Ethyl 2-((3-methoxybenzyl)thio)acetate (3i): The product was isolated as a colorless oil. Yield: 0.088 g (74%). ^1H NMR ($CDCl_3$, 400 MHz): *δ* (ppm) 7.24-7.20 (m, 1H), 6.93-6.88 (m, 2H), 6.81-6.78 (m, 1H), 4.18 (q, *J* = 7.1 Hz, 2H), 3.80 (s, 5H), 3.08 (s, 2H), 1.29 (t, *J* = 7.1 Hz, 3H). $^{13}C\{^1H\}$ NMR ($CDCl_3$, 100 MHz): *δ* (ppm) 170.3, 159.7, 138.8, 129.4, 121.5, 114.5, 112.9, 61.2, 55.1, 36.3, 32.3, 14.1. MS (EI, 70 eV; m/z (relative intensity)): 240 (50), 153 (100), 122 (17), 121 (92), 91 (34). HRMS: calcd for $C_{12}H_{16}O_3S$ (ESI-TOF, $[M+Na]^+$), 263.0712; found 263.0709.

Ethyl 2-(butylthio)acetate (3j):[6] The product was isolated as a yellow oil. Yield: 0.035 g (40%). ^1H NMR ($CDCl_3$, 400 MHz): *δ* (ppm) 4.19 (q, *J* = 7.1 Hz, 2H), 3.20 (s, 2H), 2.64 (t, *J* = 7.1 Hz, 2H), 1.59 (quint, *J* = 7.1 Hz, 2H), 1.42 (sext, *J* = 7.1 Hz, 2H), 1.29 (t, *J* = 7.1 Hz, 3H), 0.92 (t, *J* = 7.1 Hz, 3H). $^{13}C\{^1H\}$ NMR ($CDCl_3$, 100 MHz): *δ* (ppm) 170.6, 61.2, 33.7, 32.4, 31.1, 21.8, 14.1, 13.6. MS (EI, 70 eV; m/z (relative intensity)): 176 (42), 89 (57), 88 (87), 61 (100), 55 (45). HRMS: calcd for $C_8H_{16}O_2S$ (ESI-TOF, $[M+Na]^+$), 199.0763; found 199.0760.

Methyl 2-(benzylthio)acetate (3k):[7] The product was isolated as a colorless oil. Yield: 0.029 g (35%). ^1H NMR ($CDCl_3$, 400 MHz): *δ* (ppm) 7.35-7.31 (m, 5H), 3.82 (s, 2H), 3.71 (s, 3H), 3.08 (s, 2H). ^{13}C $\{^1H\}$ NMR ($CDCl_3$, 100MHz): *δ* (ppm) 170.8, 137.2, 129.1, 128.5, 127.2, 52.3, 36.4, 32.1. MS (EI, 70 eV; m/z (relative intensity)): 196 (18), 123 (82), 92 (8), 91 (100), 65 (18). HRMS: calcd for $C_{10}H_{12}O_2S$ (ESI-TOF, $[M+Na]^+$), 219.0450; found 219.0446.

Octyl 2-(benzylthio)acetate (3l): The product was isolated as a yellow oil. Yield: 0.064 g (44%). ^1H NMR ($CDCl_3$, 400 MHz): *δ* (ppm) 7.35-7.23 (m, 5H), 4.05 (q, *J* = 7.1 Hz, 2H), 3.83 (s, 2H), 3.07 (s, 2H), 1.42-1.26 (m, 9H), 0.93-0.88 (m, 6H). $^{13}C\{^1H\}$ NMR ($CDCl_3$, 100 MHz): *δ* (ppm) 171.1, 137.3, 129.1, 128.5, 127.3, 67.7, 38.8, 36.4, 33.1, 30.4, 28.9, 24.5, 22.9, 16.1, 11.8. MS (EI, 70 eV; m/z (relative intensity)): 294 (5), 123

(100), 91 (62), 71 (23), 57 (28). HRMS: calcd for $C_{17}H_{26}O_2S$ (ESI-TOF, $[M+Na]^+$), 317.1546; found 317.1545.

Cyclohexyl 2-(benzylthio)acetate (3m): The product was isolated as a yellow oil. Yield: 0.056 g (42%). ^{1}H NMR ($CDCl_3$, 400 MHz): *δ* (ppm) 7.35-7.23 (m, 5H), 4.84-4.77 (m, 1H), 3.83 (s, 2H), 3.05 (s, 2H), 1.90-1.73 (m, 4H), 1.51-1.22 (m, 6H). ^{13}C{^{1}H} NMR ($CDCl_3$, 100 MHz): *δ* (ppm) 170.4, 137.3, 129.1, 128.5, 127.2, 73.7, 36.3, 32.6, 31.5, 25.3, 23.7. MS (EI, 70 eV; m/z (relative intensity)): 264 (8), 182 (28), 181 (27), 123 (100), 91 (91), 55 (45). HRMS: calcd for $C_{15}H_{20}O_2S$ (ESI-TOF, $[M+Na]^+$), 287.1076, found 287.1073.

***tert*-Butyl 2-(benzylthio)acetate (3n):**[8] The product was isolated as a colorless oil. Yield: 0.081 g (70%). ^{1}H NMR ($CDCl_3$, 400 MHz): *δ* (ppm) 7.35-7.21 (m, 5H), 3.82 (s, 2H), 2.98 (s, 2H), 1.49 (s, 9H). ^{13}C{^{1}H} NMR ($CDCl_3$, 100 MHz): *δ* (ppm) 169.5, 137.4, 129.1, 128.4, 127.1, 81.5, 36.1, 33.5, 28.0. MS (EI, 70 eV; m/z (relative intensity)): 238 (3), 182 (30), 181 (34), 91 (90), 57 (100). HRMS: calcd for $C_{13}H_{18}O_2S$ (ESI-TOF, $[M+Na]^+$), 261,0919; found 261.0915.

Allyl 2-(benzylthio)acetate (3o): The product was isolated as a light pink oil. Yield: 0.021 g (20%). ^{1}H NMR ($CDCl_3$, 400 MHz): *δ* (ppm) 7.35-7.28 (m, 5H), 5.97-5.87 (m, 1H), 5.38-5.35 (m, 1H), 4.61 (dt, *J* = 5.8, 1.4 Hz, 2H), 3.83 (s, 2H), 3.09 (s, 2H). ^{13}C{^{1}H} NMR ($CDCl_3$, 100 MHz): *δ* (ppm) 170.0, 137.2, 131.8, 129.1, 128.5, 127.2, 118.6, 65.8, 36.4, 32.3. MS (EI, 70 eV; m/z (relative intensity)): 222 (8), 181 (14), 123 (56), 91 (100), 65 (18). HRMS: calcd for $C_{12}H_{14}O_2S$ (ESI-TOF, $[M + Na]^+$), 245.0607; found 245.0613.

Benzyl 2-(benzylthio)acetate (3q):[4] The product was isolated as a colorless oil. Yield: 0.056 g (44%). ^{1}H NMR ($CDCl_3$, 400 MHz): *δ* (ppm) 7.39–7.33 (m, 5H), 7.30–7.25 (m, 5H), 5.15 (s, 2H), 3.80 (s, 2H), 3.11 (s, 2H). ^{13}C{^{1}H} NMR ($CDCl_3$, 100 MHz): *δ* (ppm) 170.1, 137.1, 135.6, 129.1, 128.6, 128.5, 128.4, 128.3, 127.2, 67.0, 36.3, 32.3. MS (EI, 70 eV; m/z (relative intensity)): 272 (1), 181 (63), 107 (22), 92 (8), 91 (100), 65 (16). HRMS: calcd for $C_{16}H_{16}O_2S$ (ESI-TOF, $[M+Na]^+$), 295.0763, found 295.0765.

Typical procedure for the synthesis of α-thioketones 4: To a reaction tube equipped with a stir bar containing a solution of β-keto ester **1** (0.5 mmol) in toluene (3.0 mL) were added sodium *S*-organyl sulfurothioate **2** (1.0 mmol) and NaOH (1.0 mmol, 0.040 g). The tube was sealed, and the mixture was stirred at 100 °C under air for 18 h. After being cooled to room temperature, the resulting mixture was quenched with water (20 mL) and extracted with ethyl acetate (3 x 20 mL). The combined organic phases were washed with brine (20 mL), dried over anhydrous $MgSO_4$, filtered, and evaporated under reduced pressure. The residue was purified by column chromatography on silica gel with hexane/ethyl acetate (99:1) as eluent to give **4**.

1-(Benzylthio)propan-2-one (4a):[3] The product was isolated as a colorless oil. Yield: 0,0547 g (68%) / 0,073g (80%). ^{1}H NMR ($CDCl_3$, 400 MHz): *δ* (ppm) 7.34 – 7.22 (m, 5H), 3.68 (s, 2H), 3.10 (s, 2H), 2.23 (s, 3H). ^{13}C{^{1}H} NMR ($CDCl_3$, 100 MHz): *δ* (ppm) 203.5, 137.2, 129.1, 128.5, 127.2, 40.8, 36.0, 27.9. MS (EI, 70 eV; m/z (relative intensity): 180 (12), 123 (45), 122 (21), 91 (100), 65 (20). HRMS: calcd for $C_{10}H_{12}OS$ (ESI-TOF, $[M+Na]^+$), 203.0501, found 203.0507.

1-((2-Chlorobenzyl)thio)propan-2-one (4b): The product was isolated as a colorless oil. Yield: 0,0900 g (84%). ^{1}H NMR ($CDCl_3$, 400 MHz): *δ* (ppm) 7.41 – 7.30 (m, 2H), 7.25 – 7.17 (m, 2H), 3.80 (s, 2H), 3.17 (s, 2H), 2.26 (s, 3H). ^{13}C{^{1}H} NMR ($CDCl_3$, 100 MHz): *δ* (ppm) 203.6, 135.0, 134.1, 131.2, 129.9, 128.7, 126.7, 41.1, 33.6, 27.8. MS (EI, 70 eV; m/z (relative intensity)): 216 (6), 214 (16), 156 (50), 127 (34), 12 (100), 89 (22). HRMS: calcd for $C_{10}H_{11}ClOS$ (ESI-TOF, $[M+Na]^+$), 237.0111, found 237.0113.

1-((4-Chlorobenzyl)thio)propan-2-one (4c): The product was isolated as a yellow oil. Yield: 0.0480 g (45%). ^{1}H NMR ($CDCl_3$, 400 MHz): *δ* (ppm) 7.28 (d, *J* = 8.6 Hz, 2H), 7.24 (d, *J* = 8.6 Hz, 2H), 3.64 (s, 2H), 3.08 (s, 2H), 2.24 (s, 3H). ^{13}C{^{1}H} NMR ($CDCl_3$, 100 MHz): *δ* (ppm) 203.2, 135.8, 133.1, 130.5, 128.7, 40.6, 35.3, 27.9. MS (EI, 70 eV; m/z (relative intensity)): 214 (24), 157 (58), 156 (26), 127 (35), 125 (100). HRMS: calcd for $C_{10}H_{11}ClOS$ (ESI-TOF, $[M+Na]^+$); 237.0111, found 237.0114.

1-((3-(Trifluoromethyl)benzyl)thio)propan-2-one (4d): The product was isolated as a colorless oil. Yield: 0.0867 g (70%). ^{1}H NMR ($CDCl_3$, 400 MHz): *δ* (ppm) 7.59 (s, 1H),

7.54 – 7.48 (m, 2H), 7.47 – 7.39 (m, 1H), 3.73 (s, 2H), 3.10 (s, 2H), 2.25 (s, 3H). $^{13}C\{^1H\}$ NMR ($CDCl_3$, 100 MHz): *δ* (ppm) 203.0, 138.4, 132.5, 131.0 (q, *J* = 32.3 Hz), 129.0, 125.8 (q, *J* = 3.8 Hz), 124.1 (q, *J* = 3.8 Hz), 124.0 (q, *J* = 272.0 Hz), 40.6, 35.4, 27.9. ^{19}F NMR ($CDCl_3$, 376 MHz,): *δ* (ppm) - 63,1. MS (EI, 70 eV; m/z (relative intensity)): 248 (31), 191 (76), 190 (28), 159 (100), 109 (18). HRMS: calcd for $C_{11}H_{11}F_3OS$ (ESI-TOF, $[M+Na]^+$); 271.0375, found 271.0381.

1-((2-Methylbenzyl)thio)propan-2-one (4e): The product was isolated as a colorless oil. Yield: 0.0686 g (70%). ^1H NMR ($CDCl_3$, 400 MHz): *δ* (ppm) 7.20 – 7.10 (m, 4H), 3.69 (s, 2H), 3.14 (s, 2H), 2.38 (s, 3H), 2.24 (s, 3H). $^{13}C\{^1H\}$ NMR ($CDCl_3$, 100 MHz): *δ* (ppm) 203.6, 136.8, 134.8, 130.7, 130.0, 127.6, 125.8, 41.2, 34.1, 27.8, 19.0. MS (EI, 70 eV; m/z (relative intensity)): 194 (18), 137 (29), 136 (27), 135 (26), 105 (100), 104 (27). HRMS: calcd for $C_{11}H_{14}OS$ (ESI-TOF, $[M+Na]^+$), 217.0658, found 217.0659.

2-(Benzylthio)-1-phenylethan-1-one (4g):[9] The product was isolated as a yellow solid. Yield: 0.0595 g (50%), m.p. 75-77 °C. ^1H NMR ($CDCl_3$, 400 MHz): *δ* (ppm) 7.94-7.91 (m, 2H), 7.59 – 7.51 (m, 1H), 7.49 – 7.41 (m, 2H), 7.38 – 7.27 (m, 5H), 3.76 (s, 2H), 3.67 (s, 2H). $^{13}C\{^1H\}$ NMR ($CDCl_3$, 100 MHz): *δ* (ppm) 194.4, 137.3, 135.5, 133.3, 129.2, 128.7, 128.6, 128.5, 127.2, 36.1, 35.9. MS (EI, 70 eV; m/z (relative intensity)): 242 (13), 120 (70), 105 (100), 91 (43), 77 (38). HRMS: calcd for $C_{15}H_{14}OS$ (ESI-TOF, $[M+Na]^+$); 265.0658, found 265.0652.

Typical procedure for the synthesis of 6, 7 and 8: To a reaction tube equipped with a stir bar containing a solution of β-keto ester **1** (0.5 mmol) in toluene (3.0 mL) were added sodium *S*-organyl sulfurothioate **2** (1.0 mmol) and NaOH (1.0 mmol, 0.040 g). The mixture was stirred at 100 °C under air for 0.5 h. After being cooled to room temperature, the resulting mixture was quenched with water (20 mL) and extracted with ethyl acetate (3 x 20 mL). The combined organic phases were washed with brine (20 mL), dried over anhydrous $MgSO_4$, filtered, and evaporated under reduced pressure. The residue was purified by column chromatography on silica gel with hexane/ethyl acetate (99:1) as eluent to give **6**, **7** or **8**.

(*E*)-3-(Benzylthio)-4-hydroxypent-3-en-2-one (6):[10] The product was isolated as white solid. Yield: 0.0953 g (86%), m.p. 52-54 °C. ^{1}H NMR ($CDCl_3$, 400 MHz): *δ* (ppm) 17.17 (s, 1H), 7.32 – 7.21 (m, 3H), 7.11 (dd, *J* = 7.9, 1.6 Hz, 2H), 3.62 (s, 2H), 2.11 (s, 6H). ^{13}C{^{1}H} NMR ($CDCl_3$, 100 MHz): *δ* (ppm) 198.2, 137.7, 129.1, 128.6, 127.1, 103.2, 41.1, 24.0. MS (EI, 70 eV; m/z (relative intensity)): 222 (21), 180 (3), 92 (9), 91 (100), 65 (13). HRMS: calcd for $C_{12}H_{14}O_2S$ (ESI-TOF, $[M+Na]^+$), 245.0607; found, 245.0606.

Mixture of keto-enol tautomers (7):[10] The products were isolated as a colorless oil. Yield: 0.0881 g (70%). Enol: ^{1}H NMR ($CDCl_3$, 400 MHz): *δ* (ppm) 13.54 (s, 1H), 7.31 – 7.23 (m, 3H), 7.15 (d, *J* = 6.5 Hz, 2H), 4.28 (q, *J* = 7.1 Hz, 2H), 3.71 (s, 2H), 1.91 (s, 3H), 1.37 (t, *J* = 7.1 Hz, 3H). ^{13}C{^{1}H} NMR ($CDCl_3$, 100 MHz): *δ* (ppm) 184.08, 173.0, 138.0, 129.0, 128.3, 126.8, 92.7, 61.4, 39.9, 20.4, 14.2. MS (EI, 70 eV; m/z (relative intensity)): 180 (10), 123 (40), 122 (18), 91 (100), 65 (18). HRMS: calcd for $C_{13}H_{16}O_3S$ (ESI-TOF, $[M+Na]^+$), 275.0712; found, 275.0719.

Mixture of keto-enol tautomers (8): The product was isolated as a light-yellow oil. Yield: 0.0625 g (53%). Enol: ^{1}H NMR ($CDCl_3$, 400 MHz): *δ* (ppm) 13.42 (d, *J* = 0.7 Hz, 1H), 7.29 – 7.22 (m, 3H), 7.12 (dd, *J* = 8.0, 1.5 Hz, 2H), 3.78 (s, 3H), 3.67 (s, 2H), 1.88 (s, 3H). ^{13}C{^{1}H} NMR ($CDCl_3$, 100 MHz): δ (ppm) 184.3, 173.5, 137.9, 129.0, 128.3, 126.9, 92.5, 52.3, 40.0, 20.4. Ketone: ^{1}H NMR ($CDCl_3$, 400 MHz): δ (ppm) 7.33 – 7.30 (m, 5H), 3.75 (s, 3H), 3.71 (s, 2H), 3.08 (s, 1H), 2.26 (s, 3H). ^{13}C{^{1}H} NMR ($CDCl_3$, 100 MHz): *δ* (ppm) 198.5, 167.7, 136.4, 129.2, 128.6, 127.5, 57.1, 52.9, 35.8, 26.8. MS (EI, 70 eV; m/z (relative intensity)): 180 (13), 123 (48), 122 (22), 91 (100), 65 (17). HRMS: calcd for $C_{12}H_{14}O_3S$ (ESI-TOF, $[M+Na]^+$), 261.0556; found, 261.0559.

Control experiments

To gain further insights into the mechanism of these methods, several control experiments were performed as shown in Schemes 1-6. Initially, **1a** (0.5 mmol) was treated with benzyl mercaptan (1.0 mmol) under standard conditions (Typical procedure for the synthesis of α-thioesters **3**), but no desired products were obtained (Scheme 1). Then, reactions of **1a** (0.5 mmol) with dibenzyl disulfide (1.0 mmol) as a sulfur source were conducted following the typical procedure for the synthesis of α-thioesters **3**. Again, under these conditions the expected products were not formed.

These experiments suggested that a thiol or a disulfide might not be crucial intermediates to these processes.

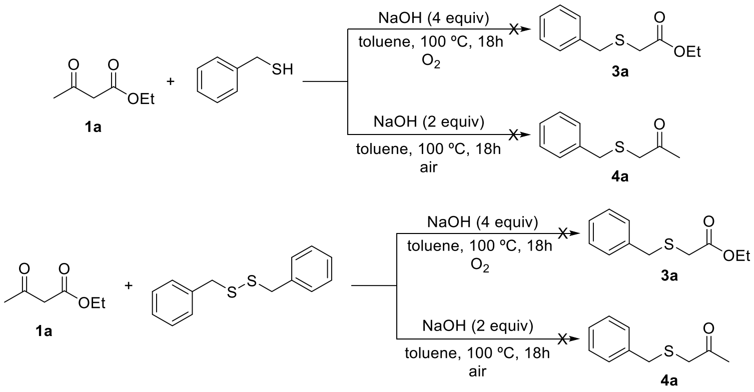

Scheme 1. Reaction of β-keto ester **1a** with benzyl mercaptan or dibenzyl disulfide under optimal conditions.

On the other hand, experiments were conducted in the presence of radical scavengers TEMPO (1.0 mmol) and hydroquinone (1.0 mmol) following the typical procedure for the synthesis of α-thioesters **3** (Scheme 2). In these cases, a mixture of products **3a** and **4a** was obtained. No radical trapping product was detected by GC-MS. Although we cannot discard the formation of radical intermediates, the results depicted in Scheme 2 implied that these reactions proceed predominantly through an ionic pathway.

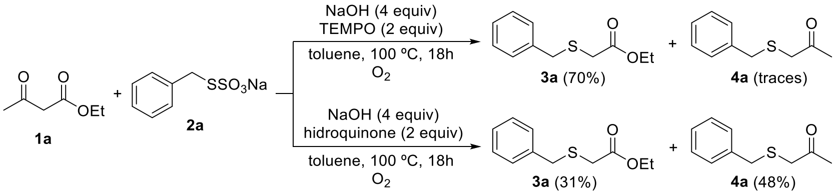

Scheme 2. Reaction of β-keto ester **1a** with Bunte salt **2a** in the presence of radical scavengers.

A close inspection in results depicted in Scheme 3 indicated that under O_2 or N_2 conditions, product **3a** was preferentially formed, while product **4a** was the main product only using lower amounts of base under air conditions. These results suggested that air humidity and residual water in the solvent might dramatically affect the selectivity of the reaction, favoring formation of **4a**.

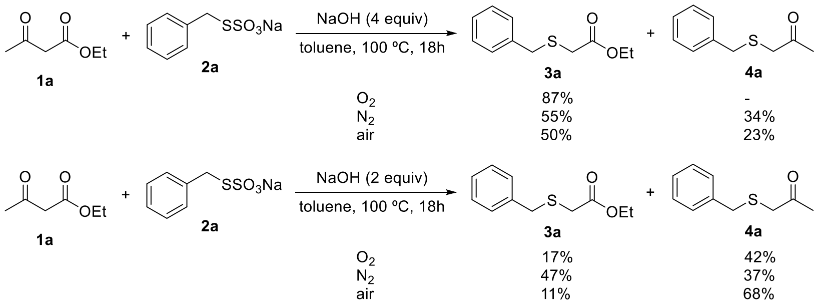

Scheme 3. Reaction of β-keto ester **1a** with Bunte salt **2a** under O_2, N_2 or air conditions.

To support this information further experiments were conducted using a mixture of toluene / water (98:2) as solvent or adding a phase-transfer catalyst (5 mol%) (PTC)[11] to the reaction system (Scheme 4) (Typical procedure for the synthesis of α-thioketone **4**). Water in this case did not benefit the selectivity nor the reaction yield, but similar results were obtained (Scheme 4). The presence of a PTC in the reaction mixture reduced both the products yield and the selectivity.

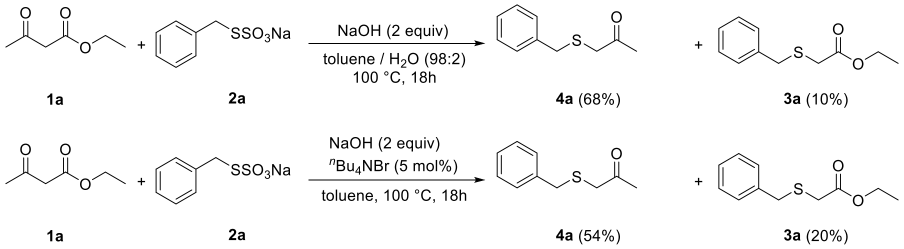

Scheme 4. Reaction of β-keto ester **1a** with Bunte salt **2a** in the presence of water or *n*-Bu_4NBr.

Next, the mixture of keto-enol tautomers **7** was subjected to the reaction conditions depicted in scheme 5 (Typical procedure for the synthesis of α-thioketone **4**). This reaction showed experimental evidence for the formation of products **3** and **4** starting from tautomers **7** in 34% and 5% isolated yield, respectively.

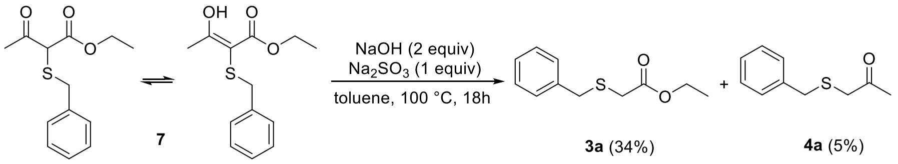

Scheme 5. Experimental evidence for the obtention of products **3** and **4** from keto-enol tautomers **7**.

Finally, when the reaction using β-keto ester **1l** (0.5 mmol) was conducted in the presence of 2 equiv of NaOH it was possible to identify from the crude mixture the formation of benzoic acid **9** by gas chromatography-mass spectrometry (GC-MS) analysis (Scheme 6). We consider that this result also supports the reaction mechanism to the formation of esters **3**.

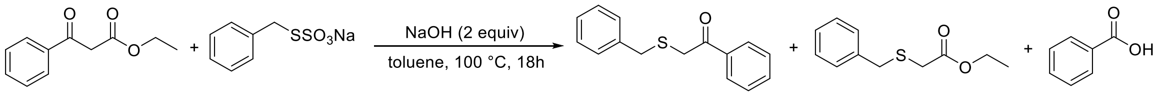

Scheme 6. Reaction of β-keto ester **1l** with Bunte salt **2a**.

3. References

[1] Reeves, J. T.; Camara, K.; Han, Z. S.; Xu, Y.; Lee, H.; Busacca, C. A.; Senanayake, C. H. *Org. Lett.* **2014**, *16*, 1196-1199.
[2] Solladié-Cavallo, A.; Bencheqroun, M. *J. Organomet. Chem.* **1991**, *403*, 159-164.
[3] He, Y-h; Li, N-b; Chen, Ji-y; Qiu, R-h; Wang, X; Xu, X-h. *Synth. Commun.* **2015**, *45*, 1817-1822.
[4] Tyagi, V.; Bonna, R. B.; Fasan, R. *Chem. Sci.*, **2015**, *6*, 2488-2494.
[5] Tamura, Y.; Tsugoshi, T.; Annoura, H.; Ishibashi, H. *Synthesis* **1984**, 326-327.
[6] Kudo, N.; Yoneda, A.; Sato, K.; Honma, T.; Sugai, S. *Chem. Pharm. Bull.* **2000**, *48*, 509-515.
[7] Audubert, C.; Lebel, H.*Org. Lett*. **2017**, *19*, 4407–4410.
[8] Zong, L.; Wang, C.; Moeljadi, A.; Ye, X.; Ganguly, R.; Li, Y.; Hirao, H.; Tan, C-h. *Nat. Commun*. **2016**, *7*, 13455-13462.
[9] Xu, X.-B.; Lin, Z.-H.; Liu, Y.; Guo, J.; He, Y. *Org. Biomol. Chem*. **2017**, *15*, 2716-2720.
[10] Tan, J.; Liang, F.; Wang, Y.; Cheng, X.; Liu, Q.; Yuan, H. *Org. Lett*. **2008**, *10*, 2485-2488.
[11] Starks, C. M. *J. Am. Chem. Soc.* **1971**, *93*, 195-199.

Copies of ^{1}H, $^{13}C\{1H\}$ and ^{19}F NMR spectra

The ^{1}H (400 MHz) and ^{13}C (100 MHz) NMR spectra of **3a** in $CDCl_3$.

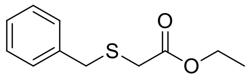

3a

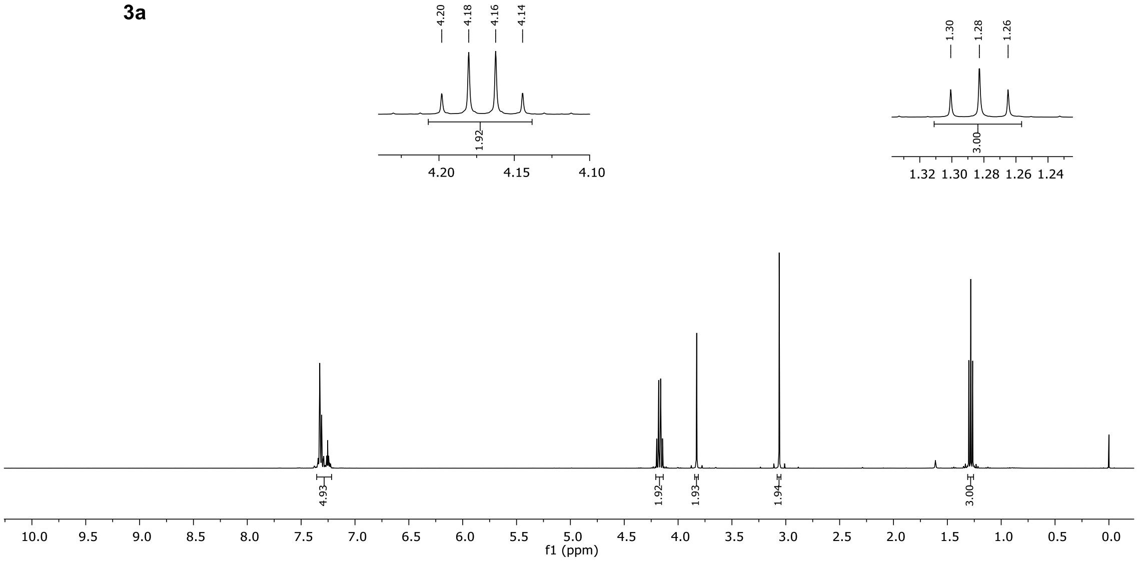

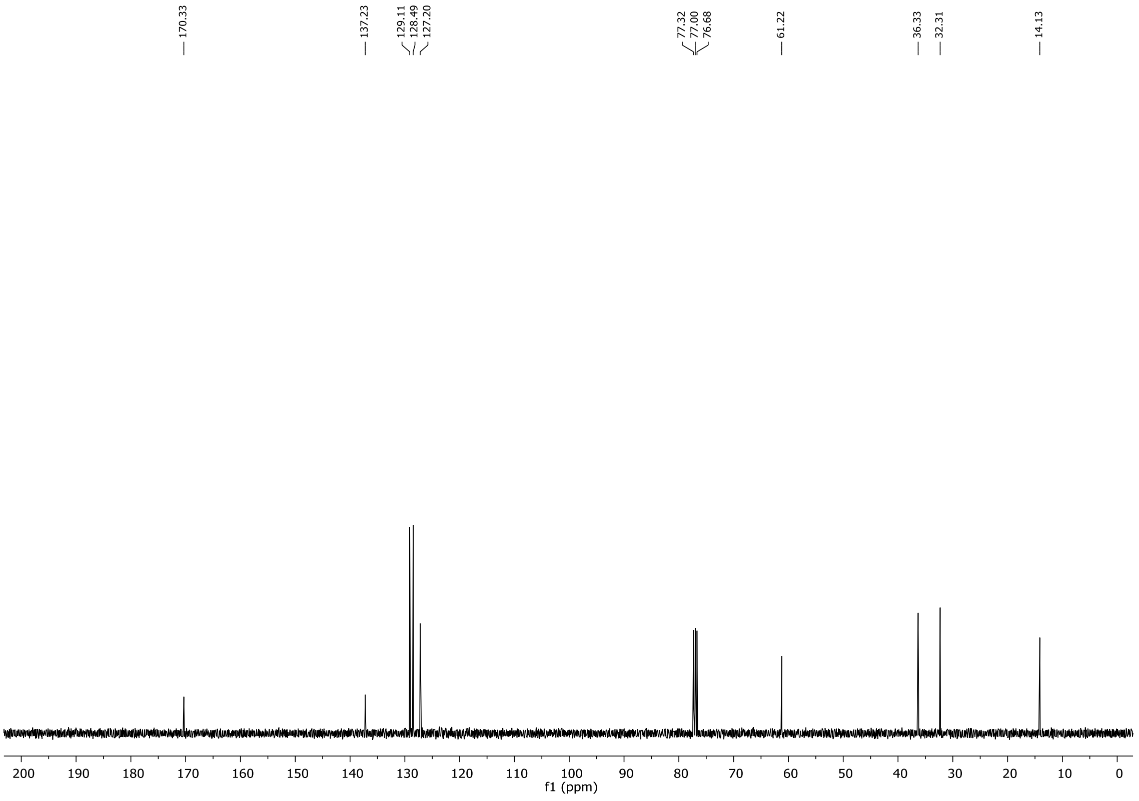

The ^{1}H (400 MHz) and ^{13}C (100 MHz) NMR spectra of **3b** in $CDCl_3$.

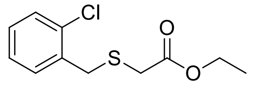

3b

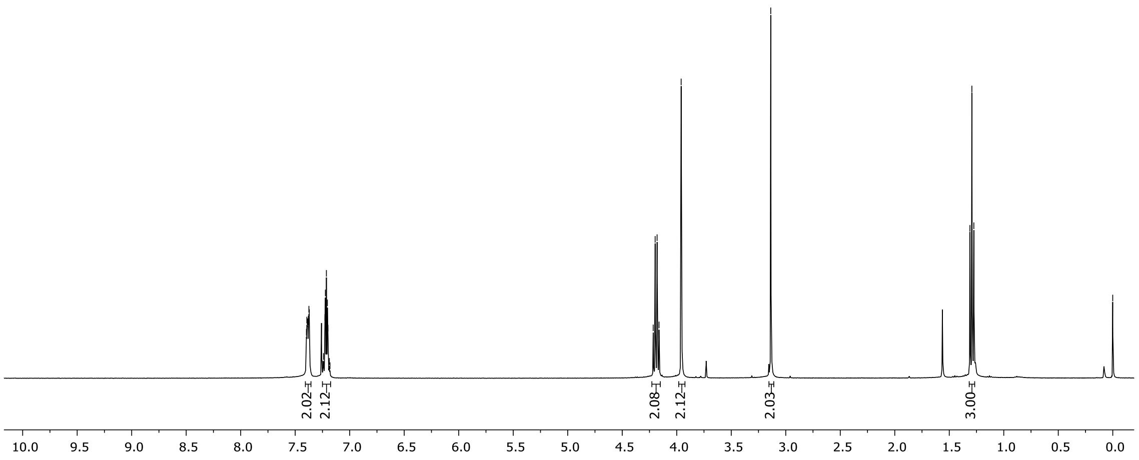

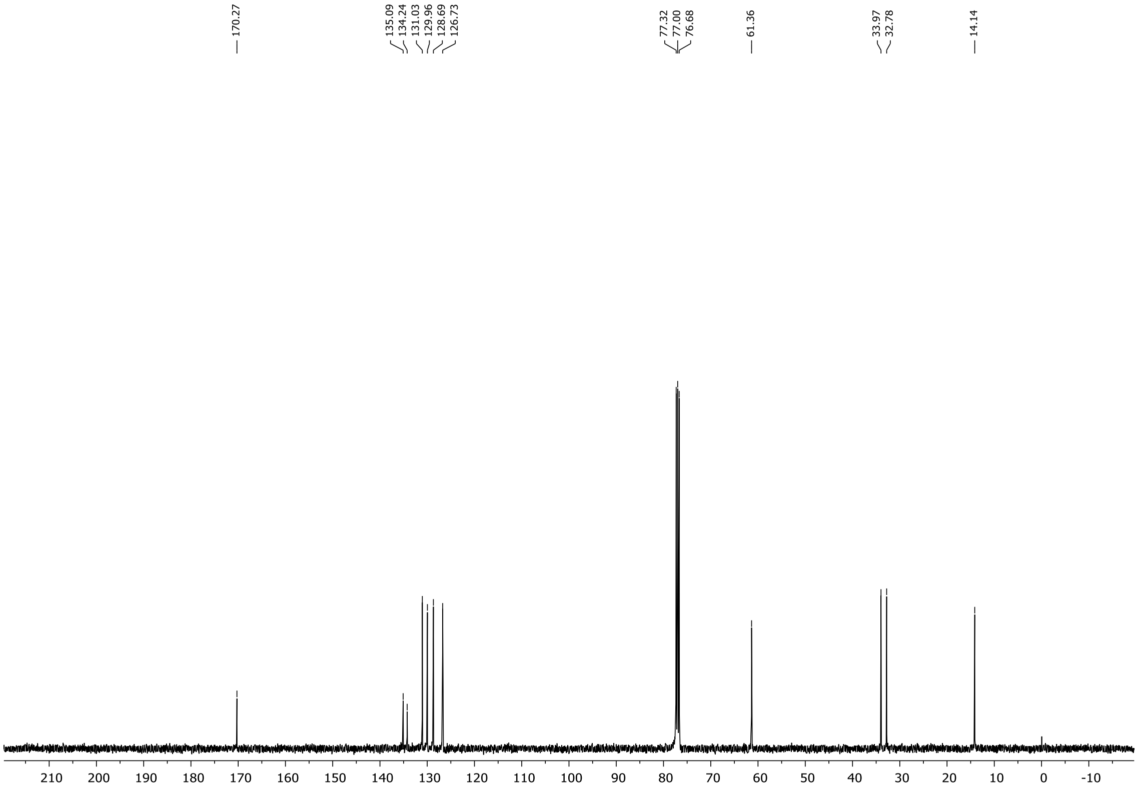

The ^{1}H (400 MHz) and ^{13}C (100 MHz) NMR spectra of **3c** in $CDCl_3$.

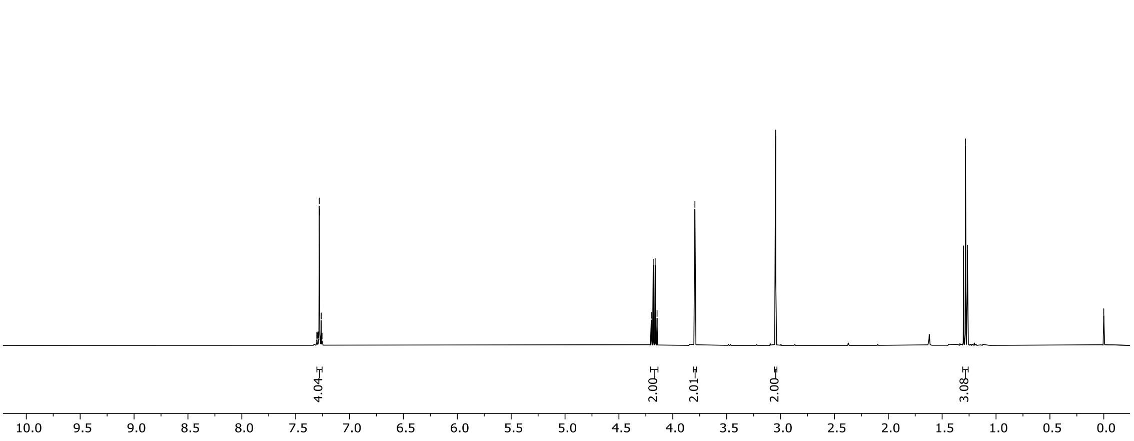

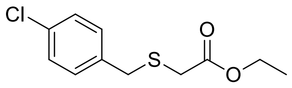

3c

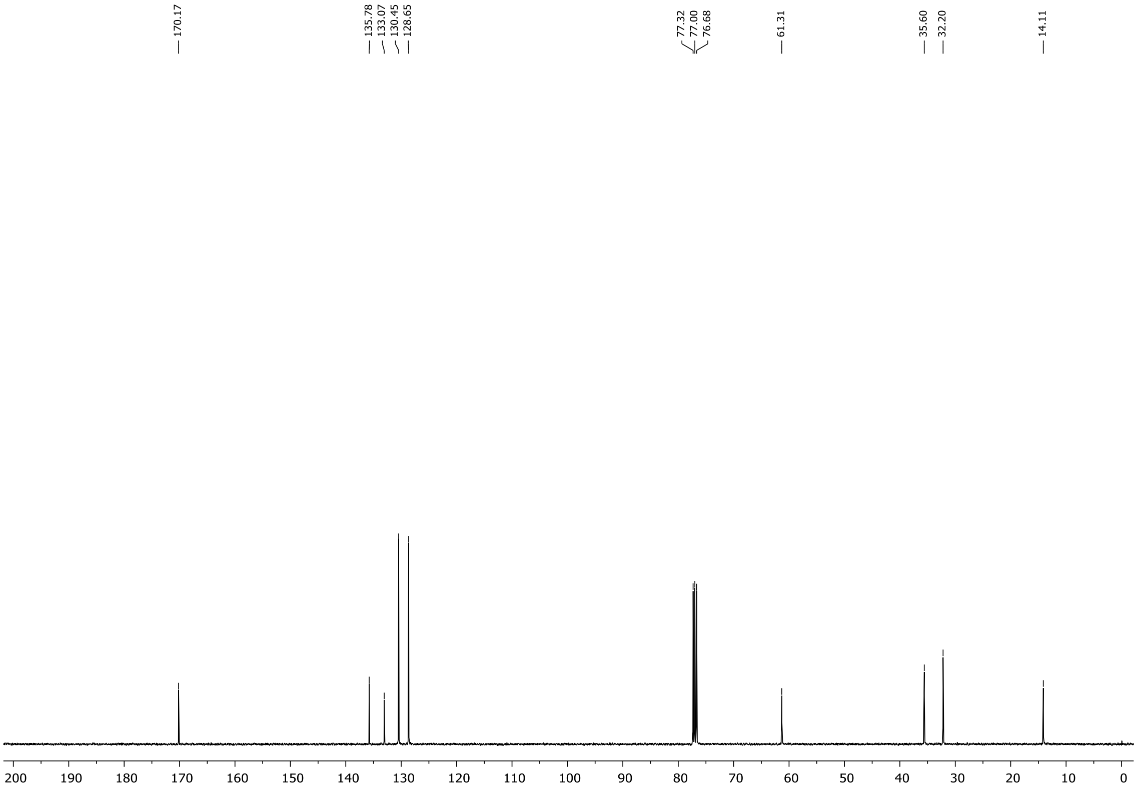

The ^{1}H (400 MHz) and ^{13}C (100 MHz) NMR spectra of **3d** in $CDCl_3$.

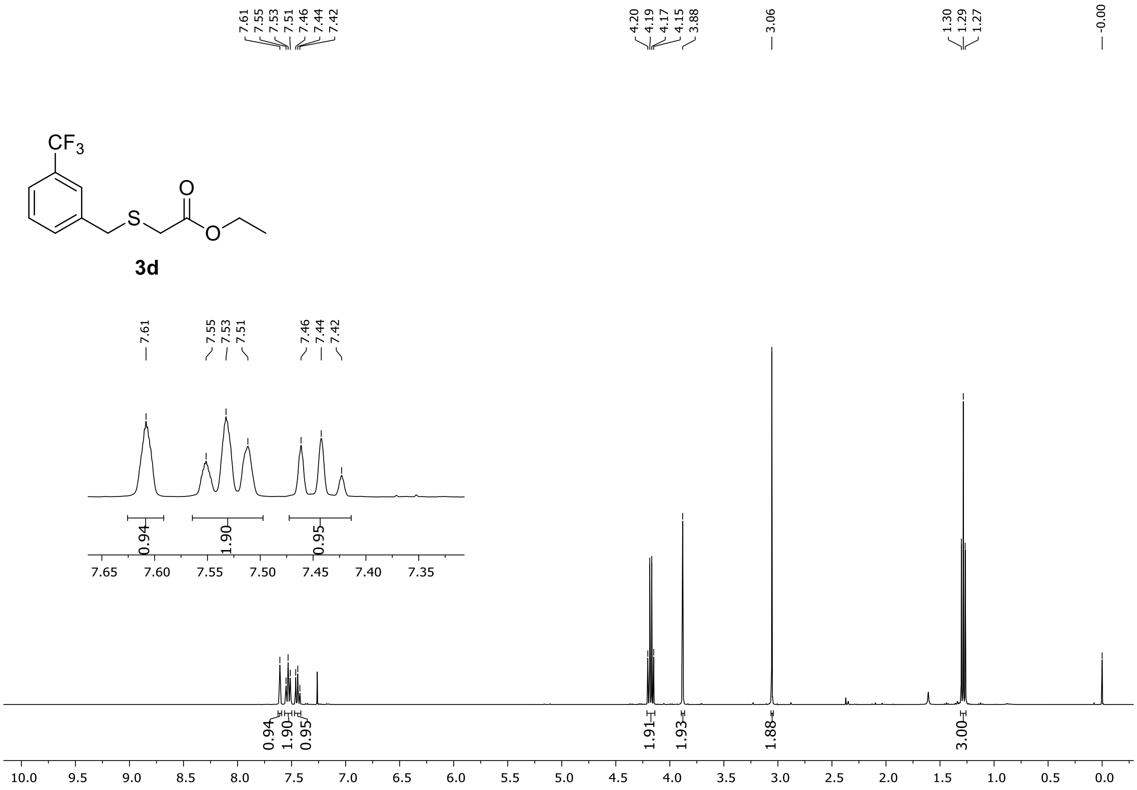

3d

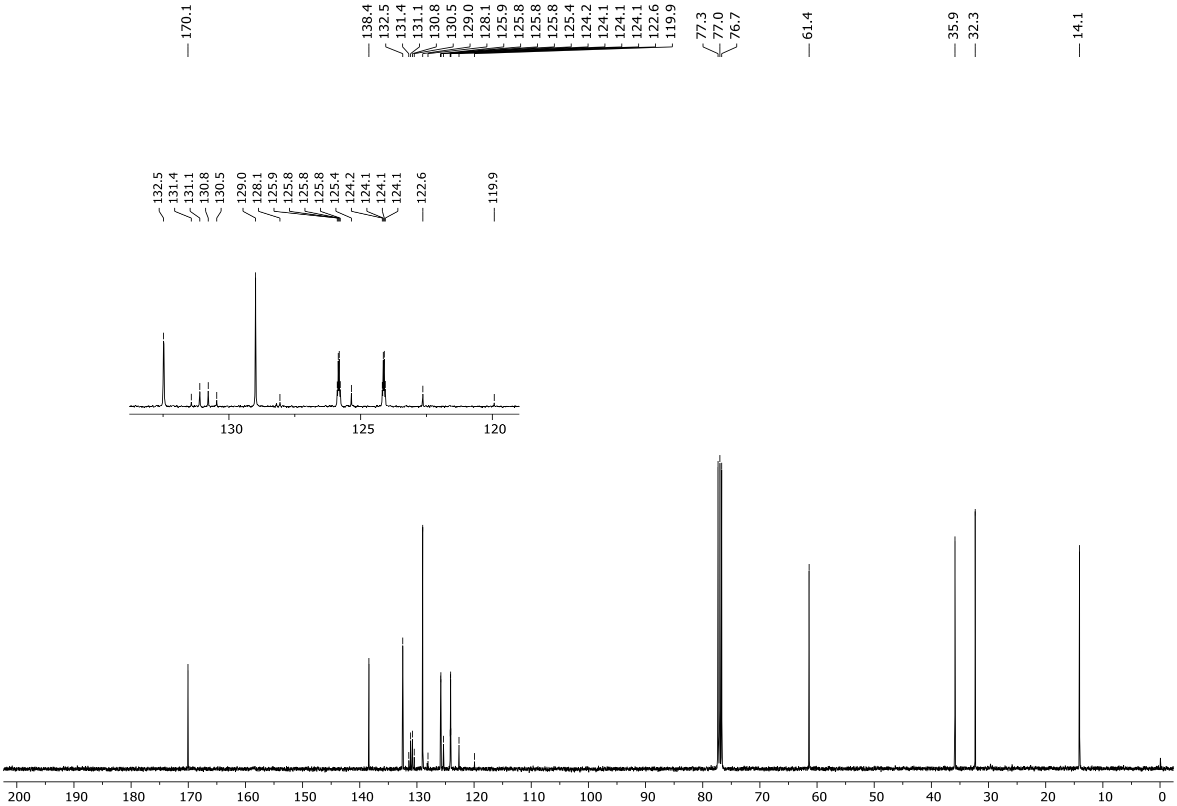

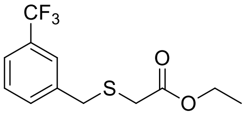
CF3
S
O
O

3d

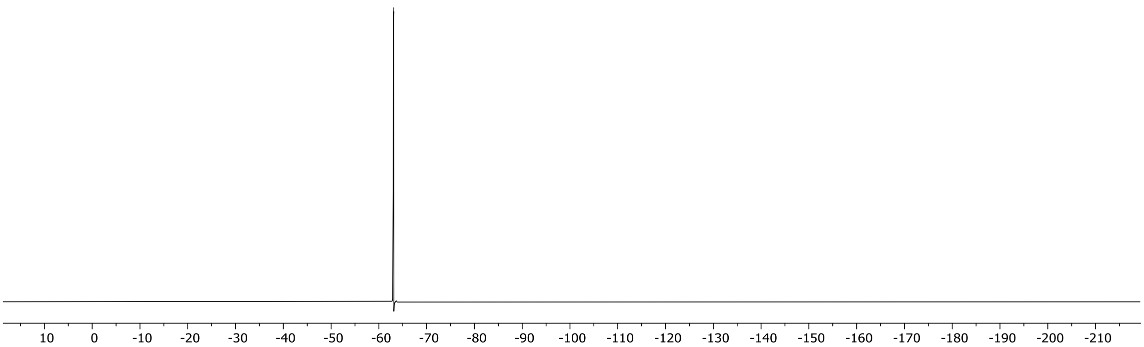
-63.13
10 0 -10 -20 -30 -40 -50 -60 -70 -80 -90 -100 -110 -120 -130 -140 -150 -160 -170 -180 -190 -200 -210

The ^{1}H (400 MHz) and ^{13}C (100 MHz) NMR spectra of **3e** in $CDCl_3$.

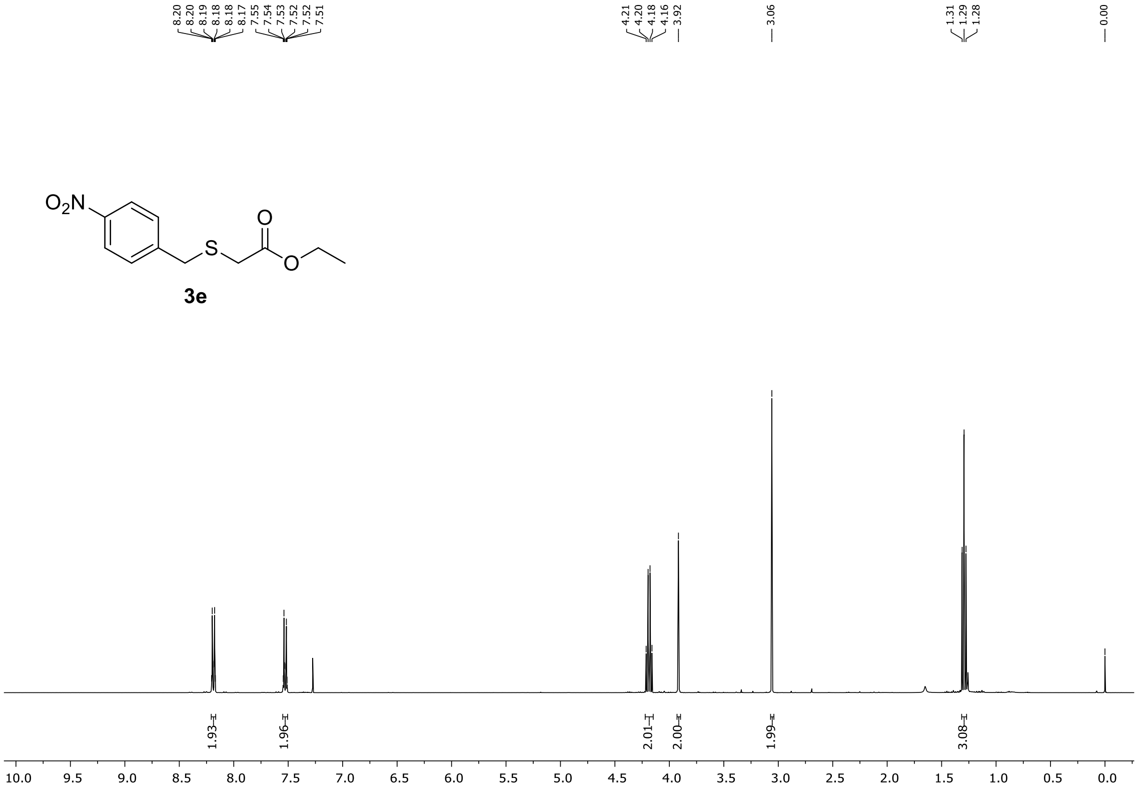

3e

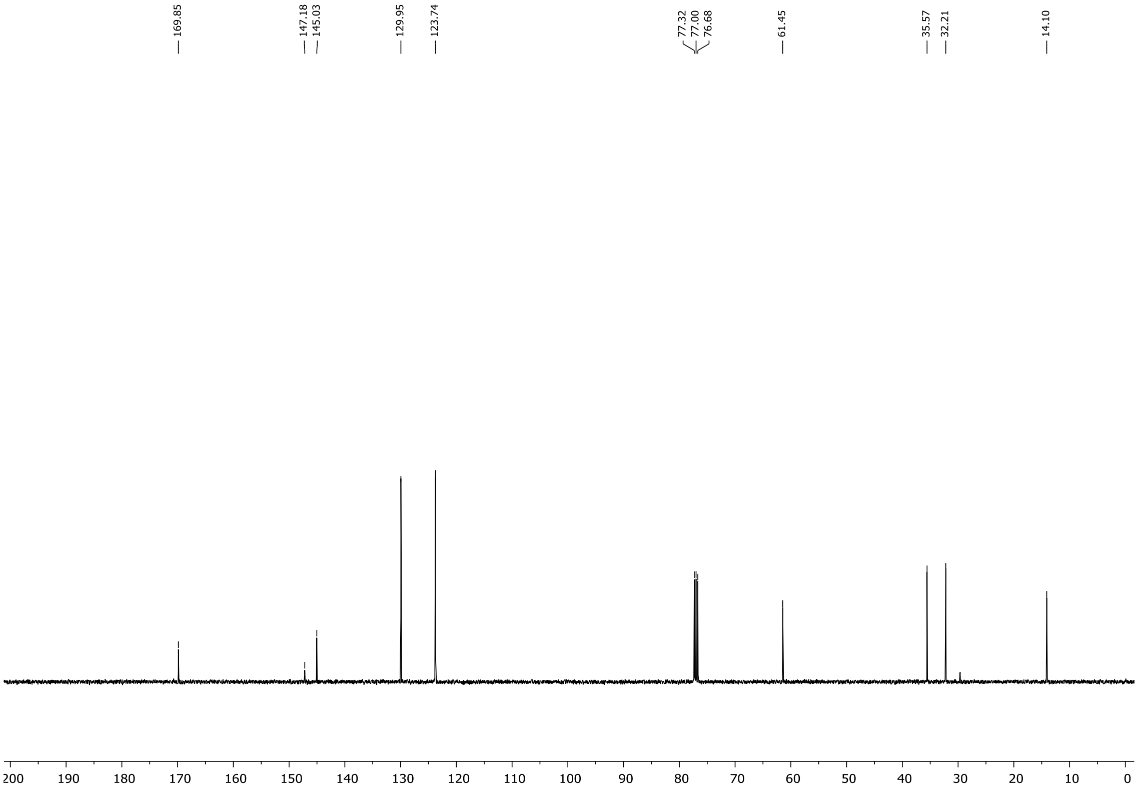

The ^{1}H (400 MHz) and ^{13}C (100 MHz) NMR spectra of **3f** in $CDCl_3$.

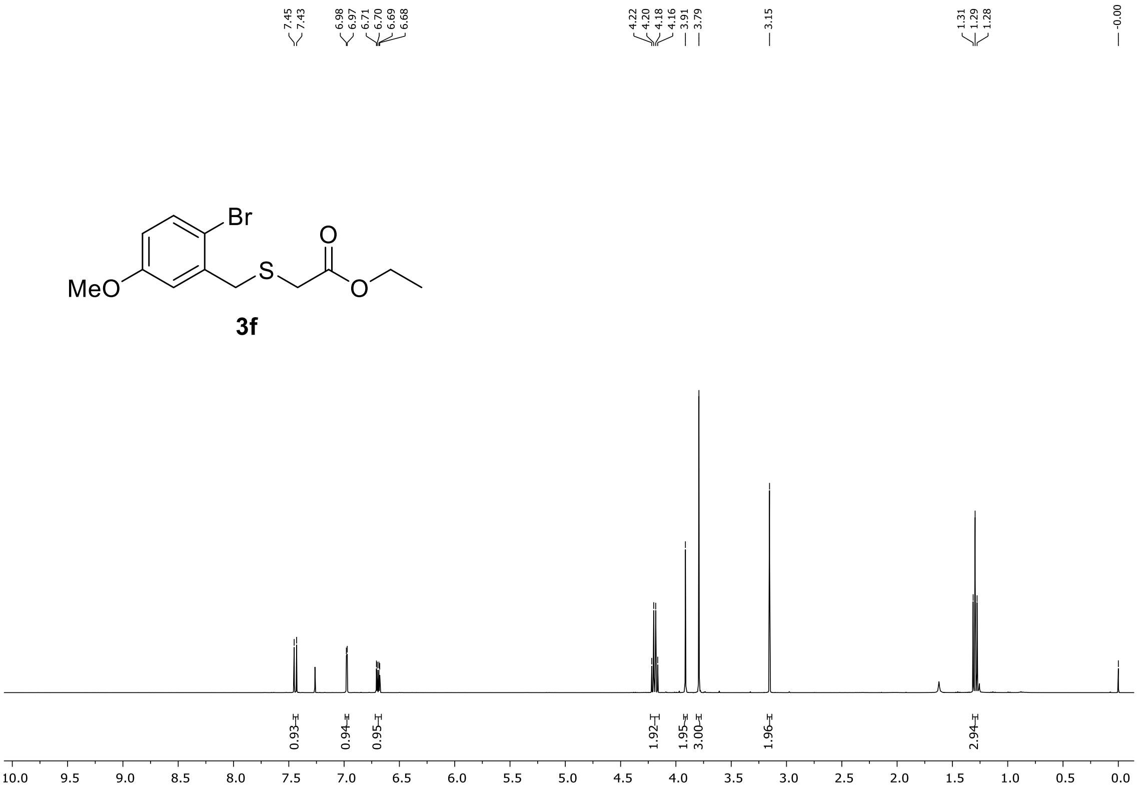

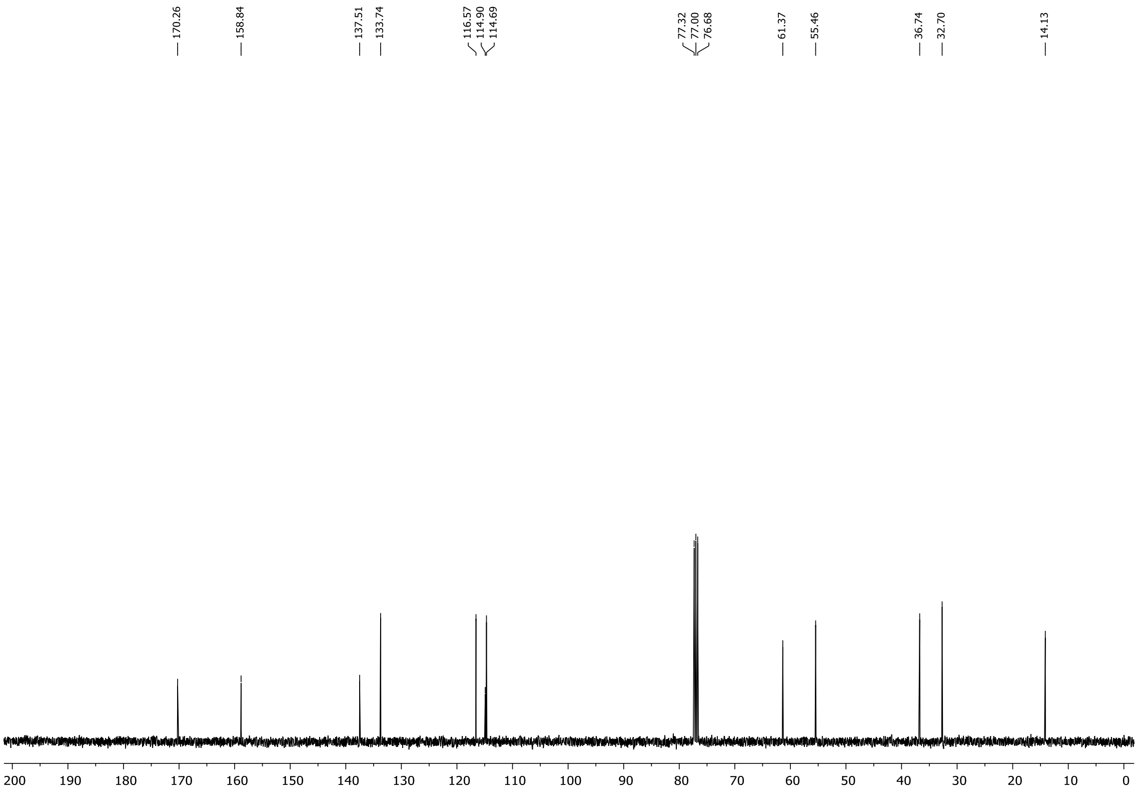

The ^{1}H (400 MHz) and ^{13}C (100 MHz) NMR spectra of **3g** in $CDCl_3$.

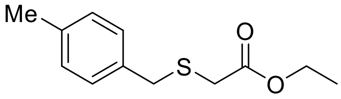

3g

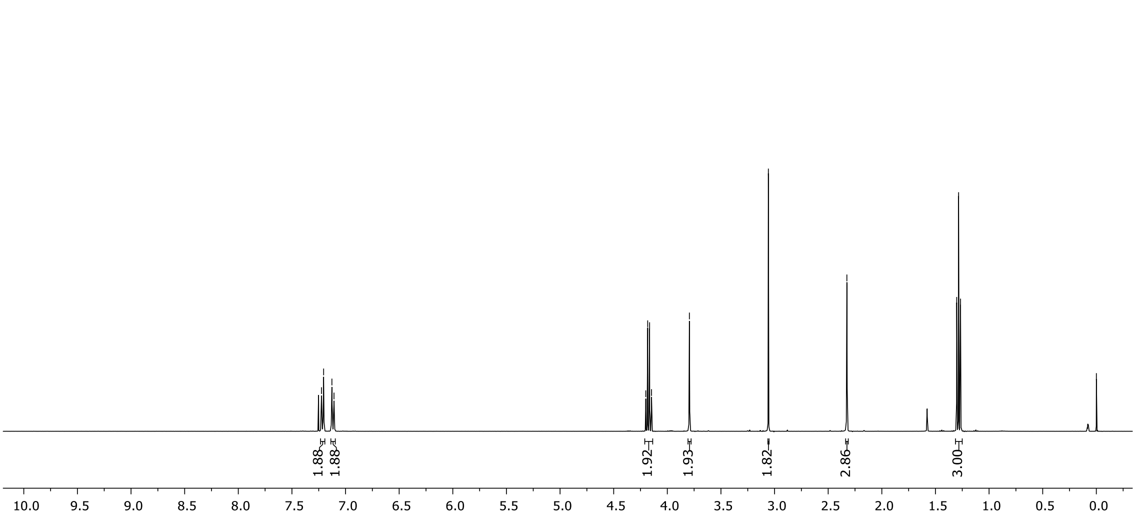

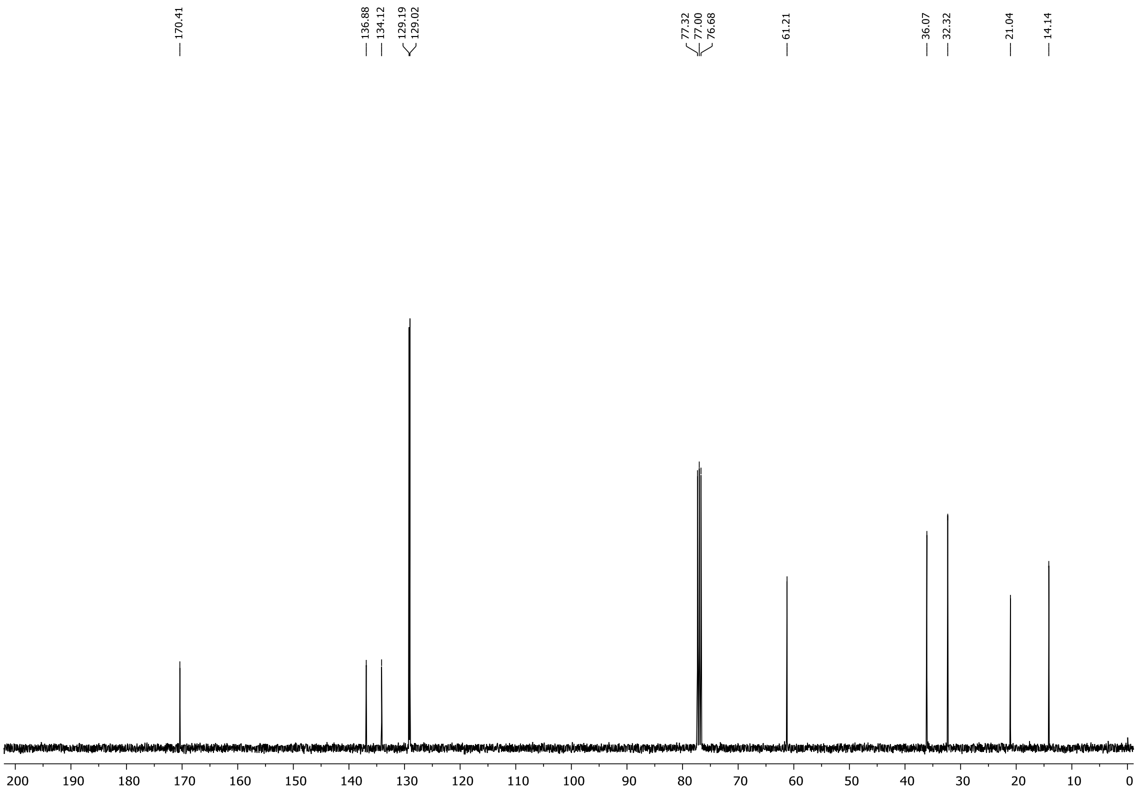

The ^{1}H (400 MHz) and ^{13}C (100 MHz) NMR spectra of **3h** in $CDCl_3$.

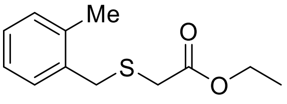

3h

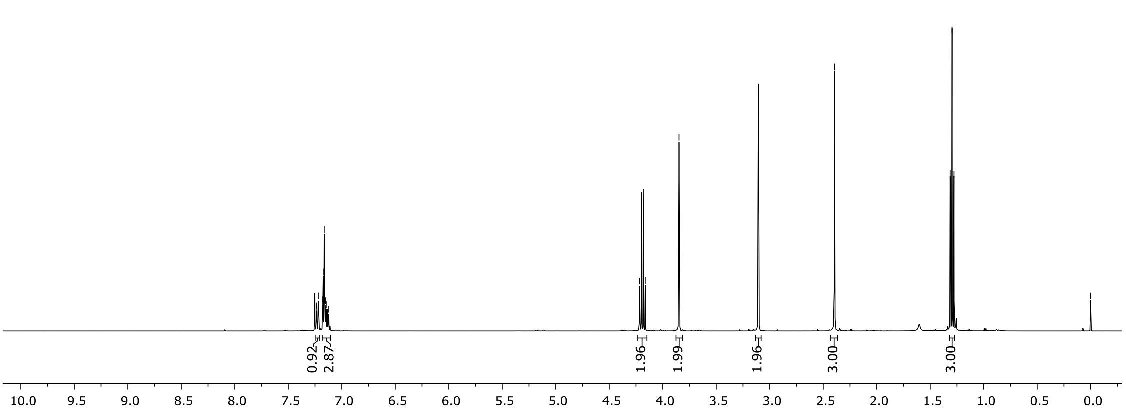

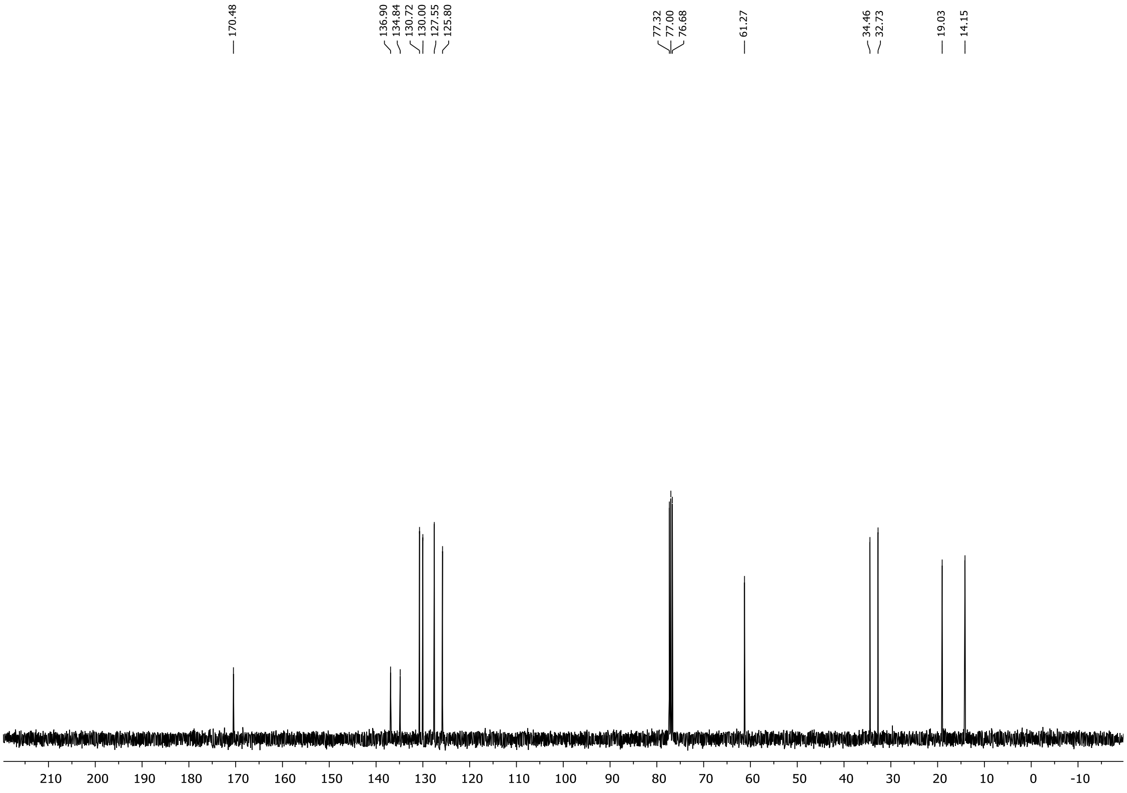

The ^{1}H (400 MHz) and ^{13}C (100 MHz) NMR spectra of **3i** in $CDCl_3$.

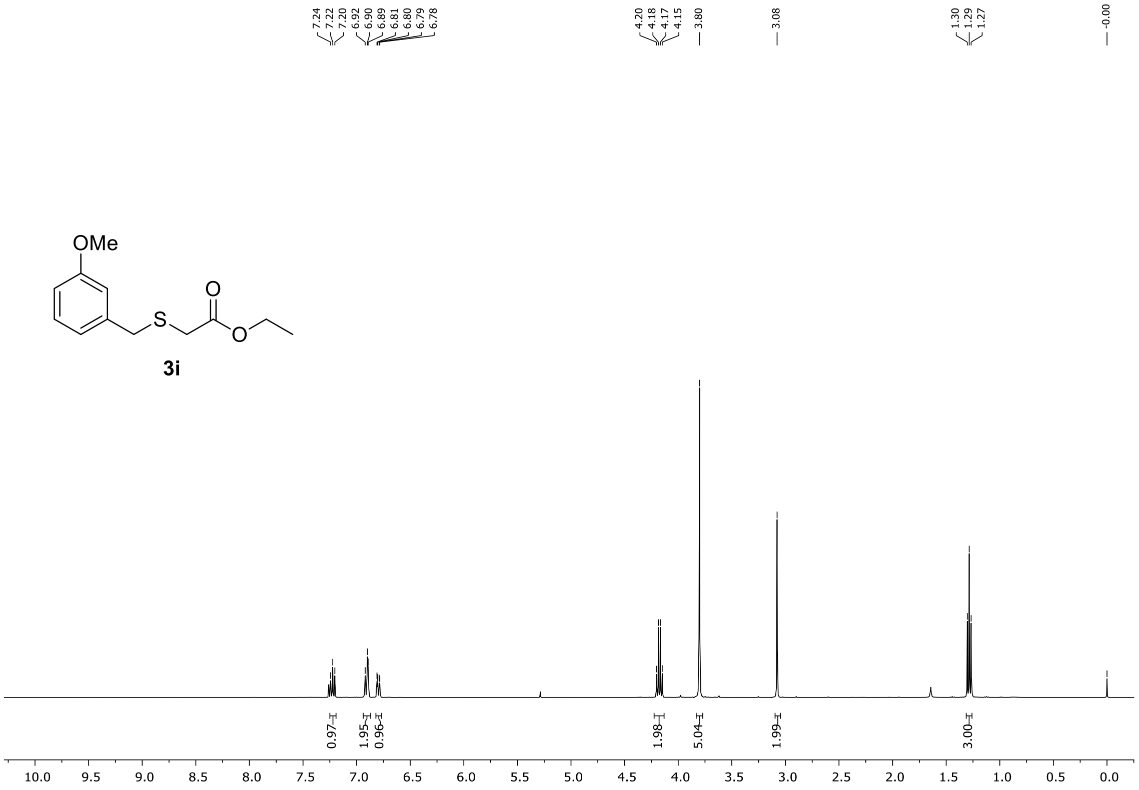

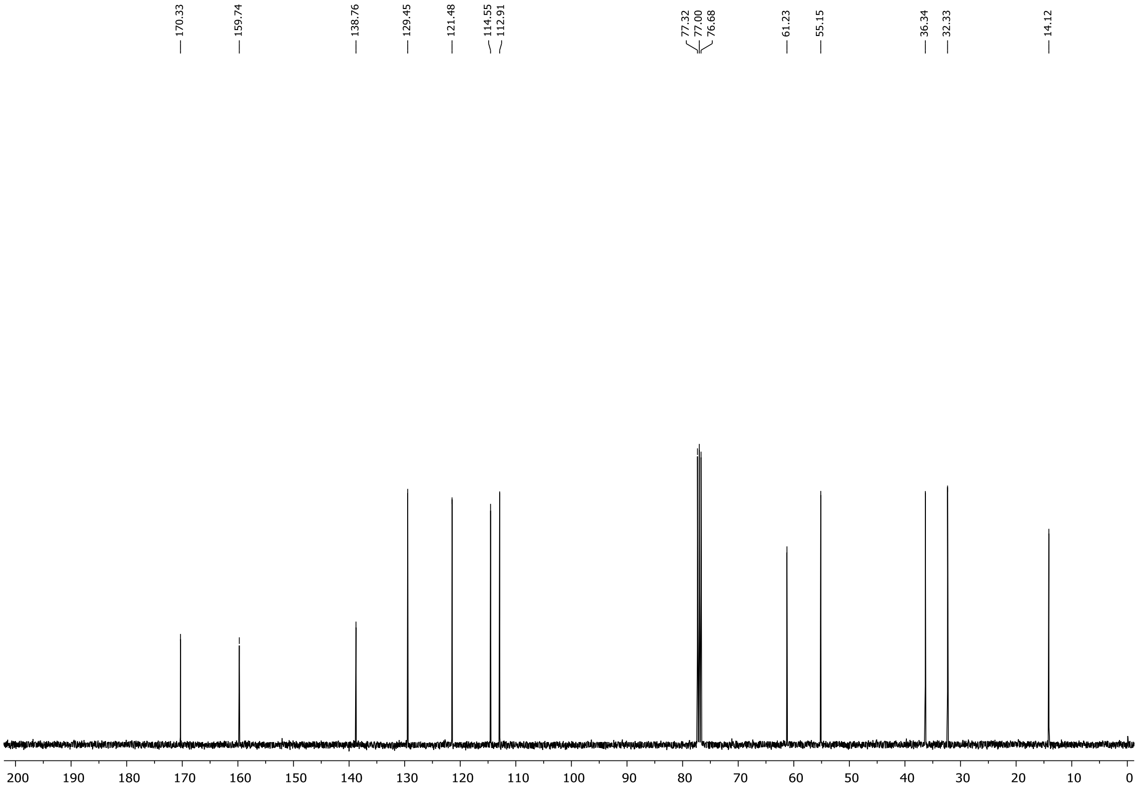

The ^{1}H (400 MHz) and ^{13}C (100 MHz) NMR spectra of **3j** in $CDCl_3$.

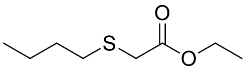

3j

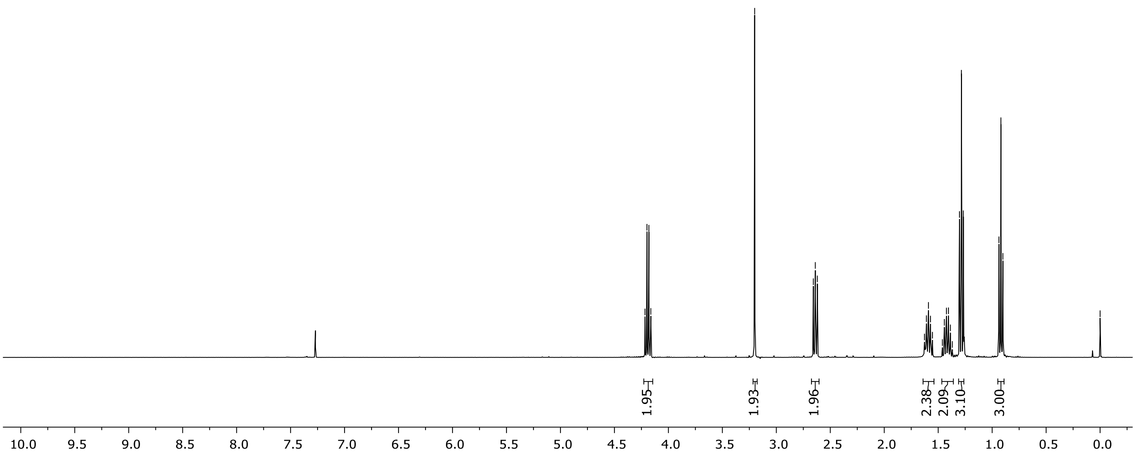

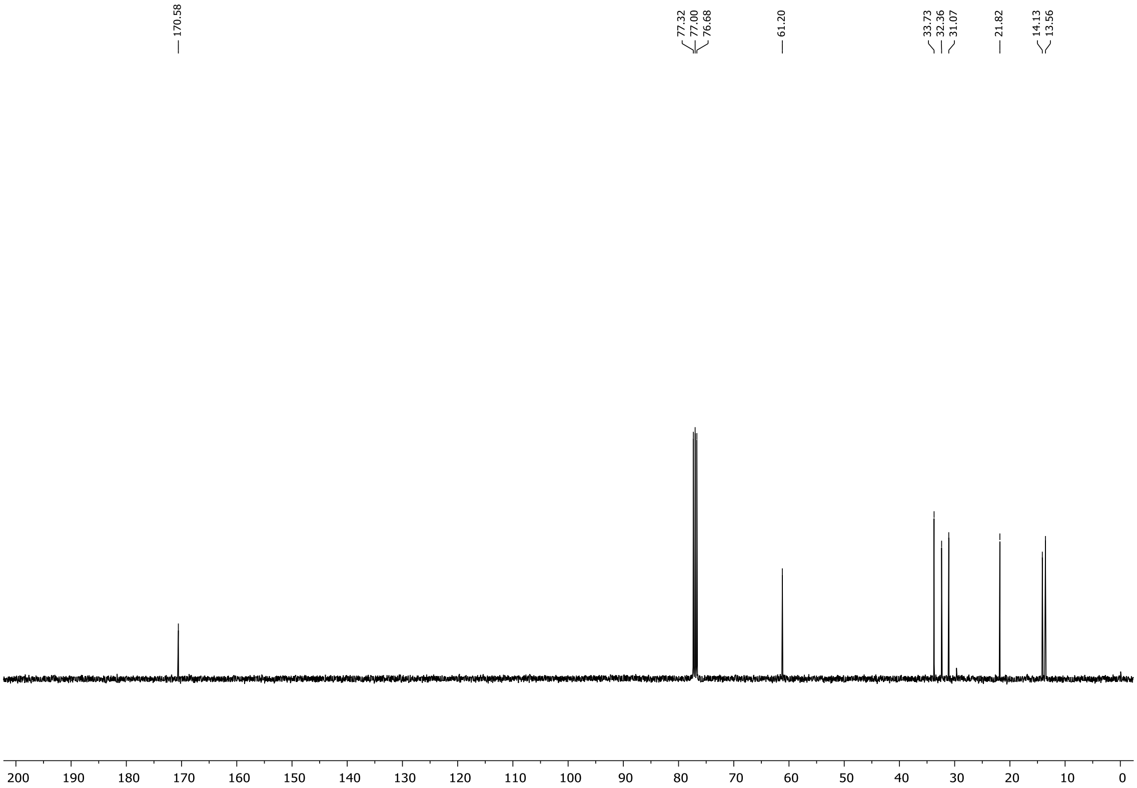

The ^{1}H (400 MHz) and ^{13}C (100 MHz) NMR spectra of **3k** in $CDCl_3$.

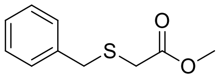

3k

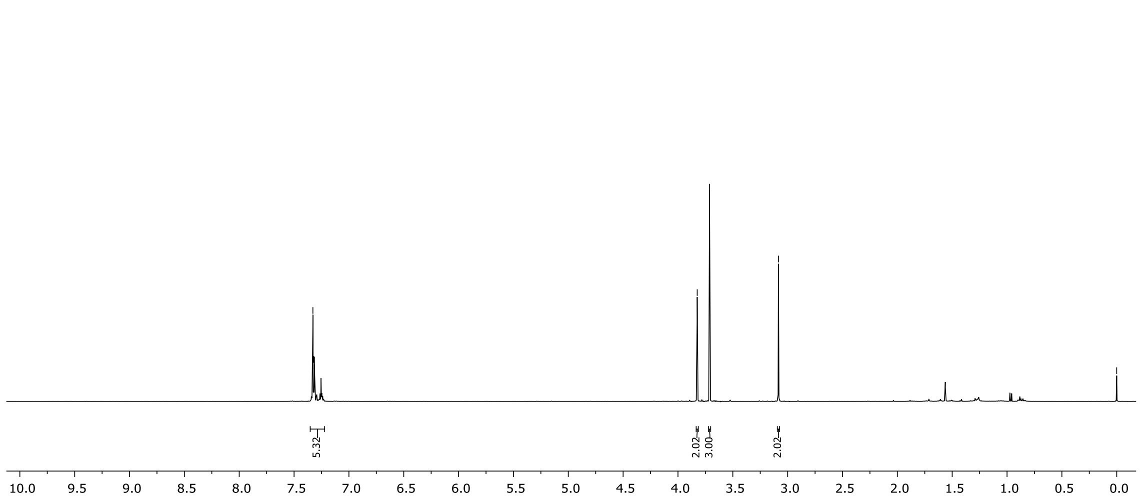

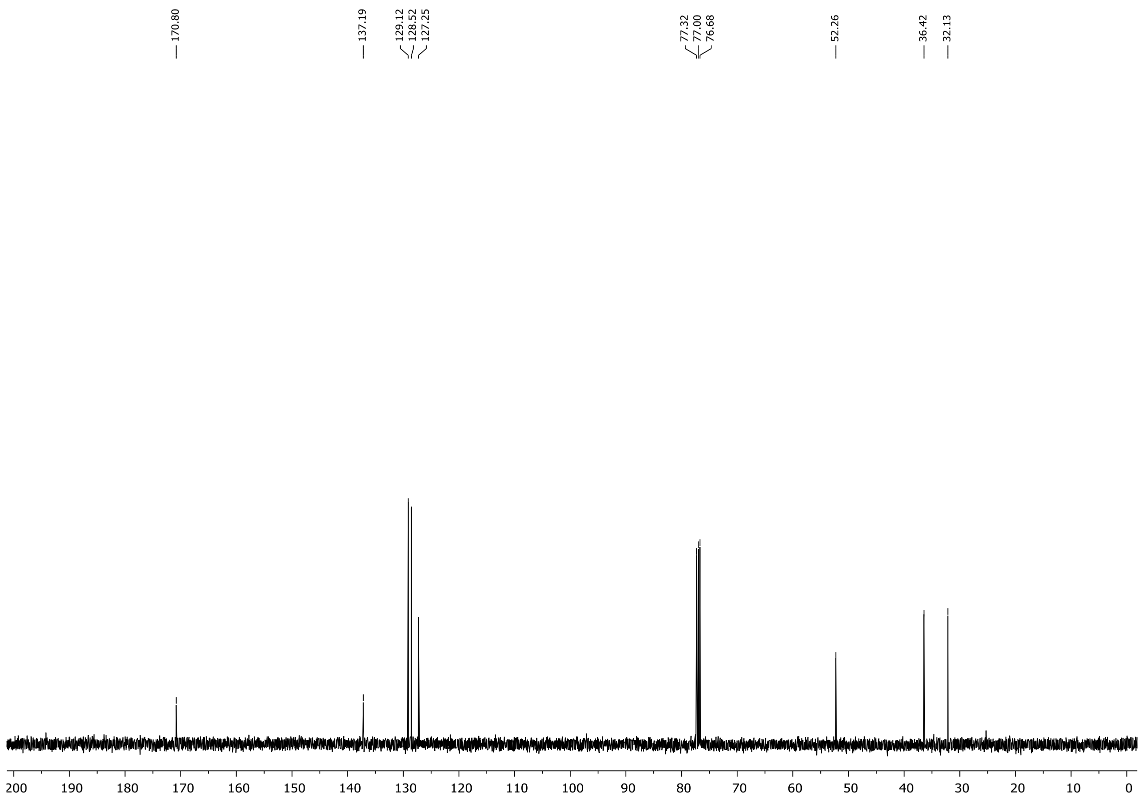

The ^{1}H (400 MHz) and ^{13}C (100 MHz) NMR spectra of **3l** in $CDCl_3$.

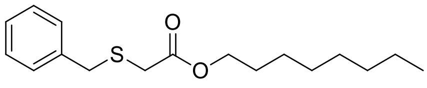

3l

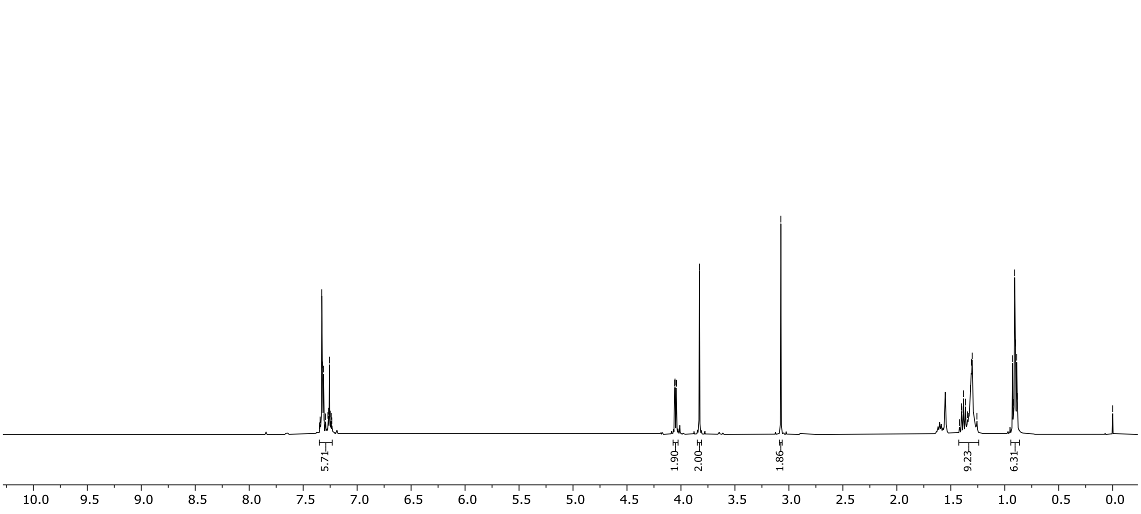

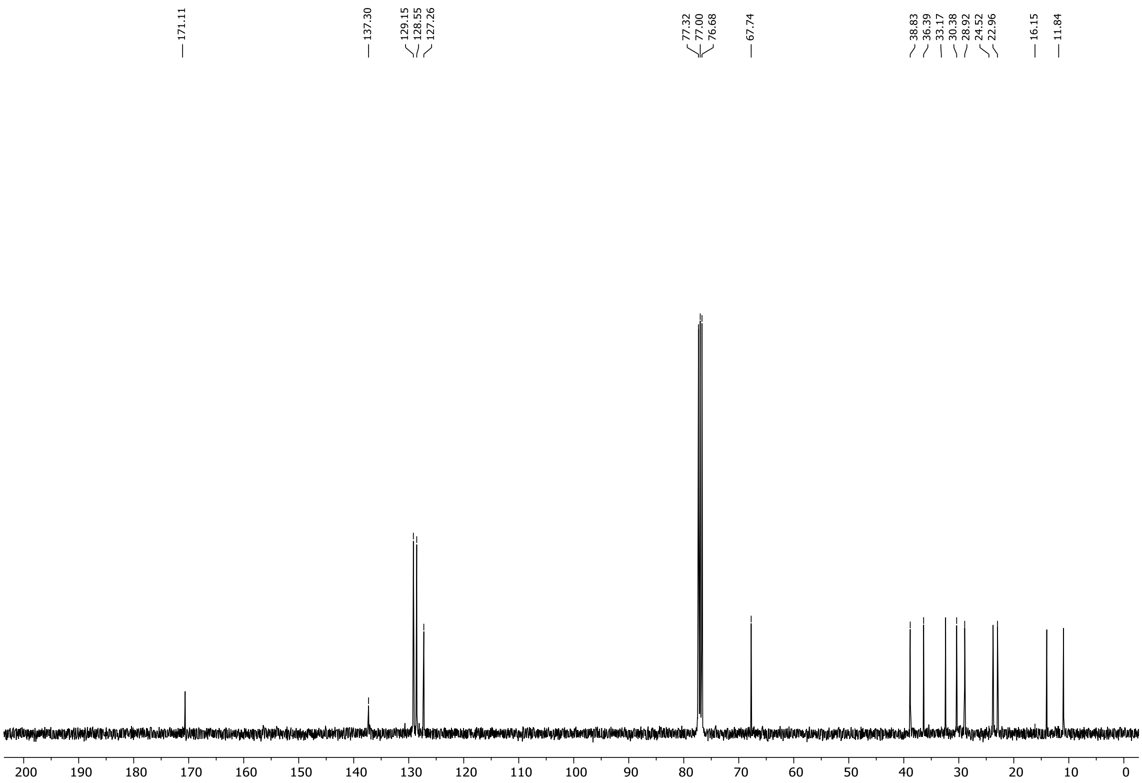

The ^{1}H (400 MHz) and ^{13}C (100 MHz) NMR spectra of **3m** in $CDCl_3$.

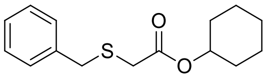

3m

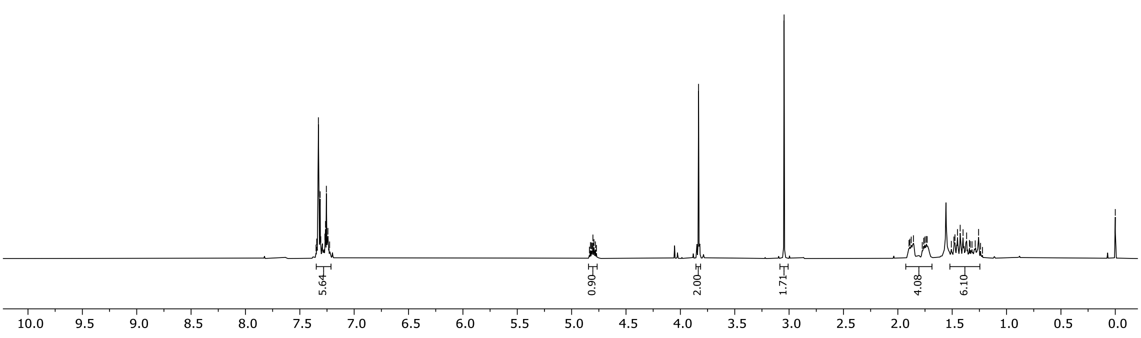

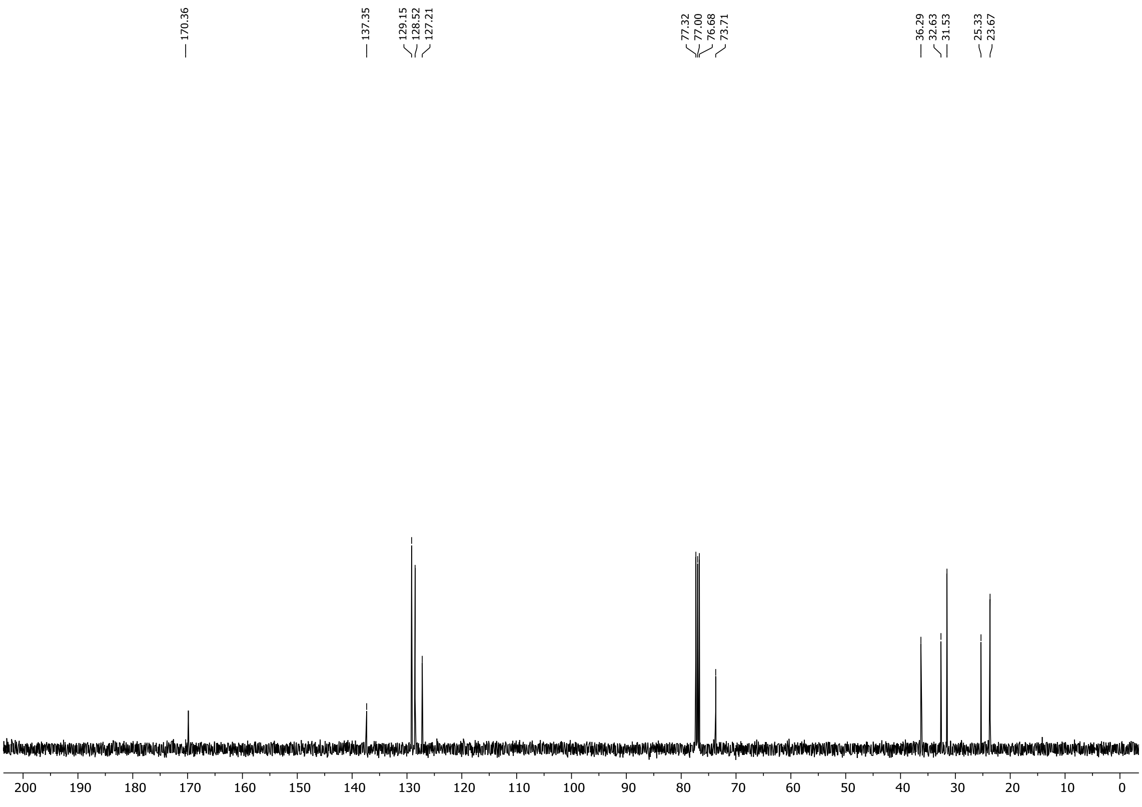

The ^{1}H (400 MHz) and ^{13}C (100 MHz) NMR spectra of **3n** in $CDCl_3$.

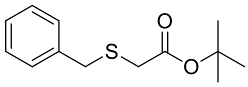

3n

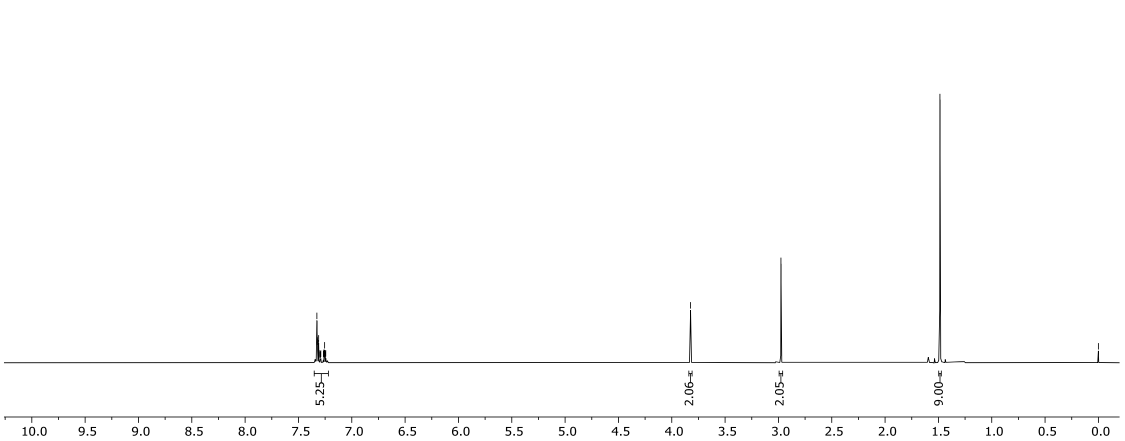

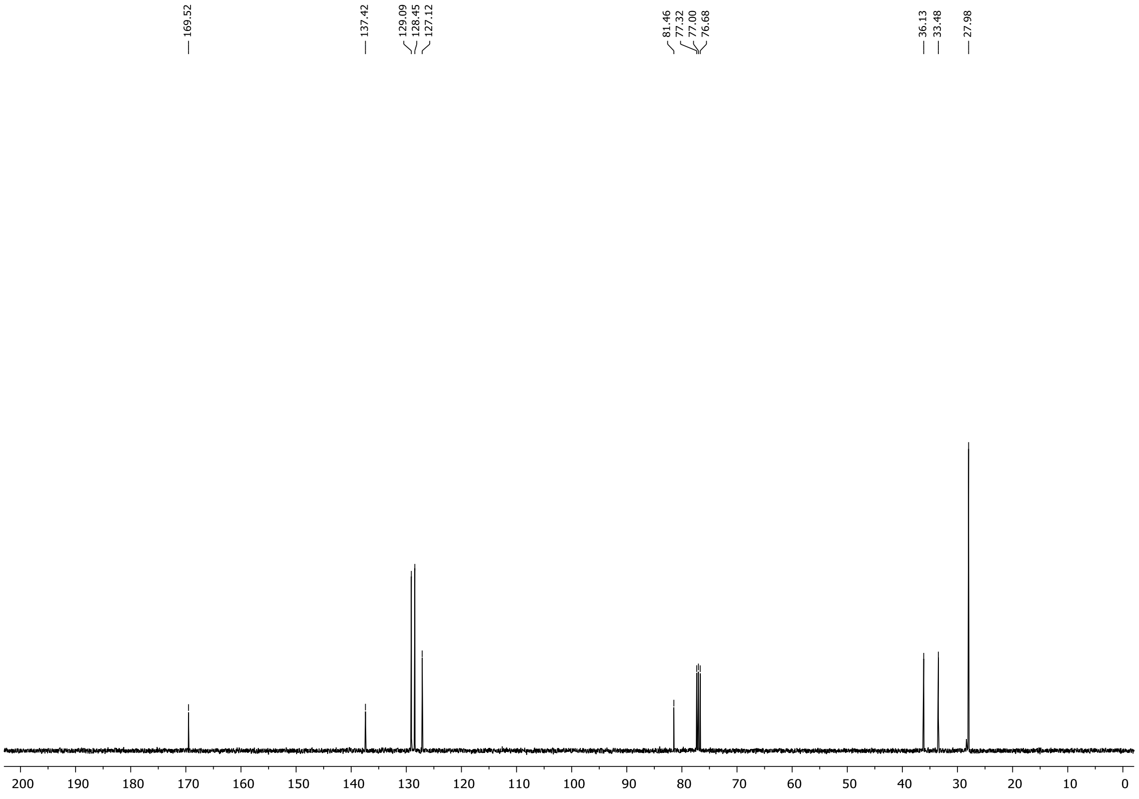

The ^{1}H (400 MHz) and ^{13}C (100 MHz) NMR spectra of **3o** in $CDCl_3$.

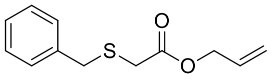

3o

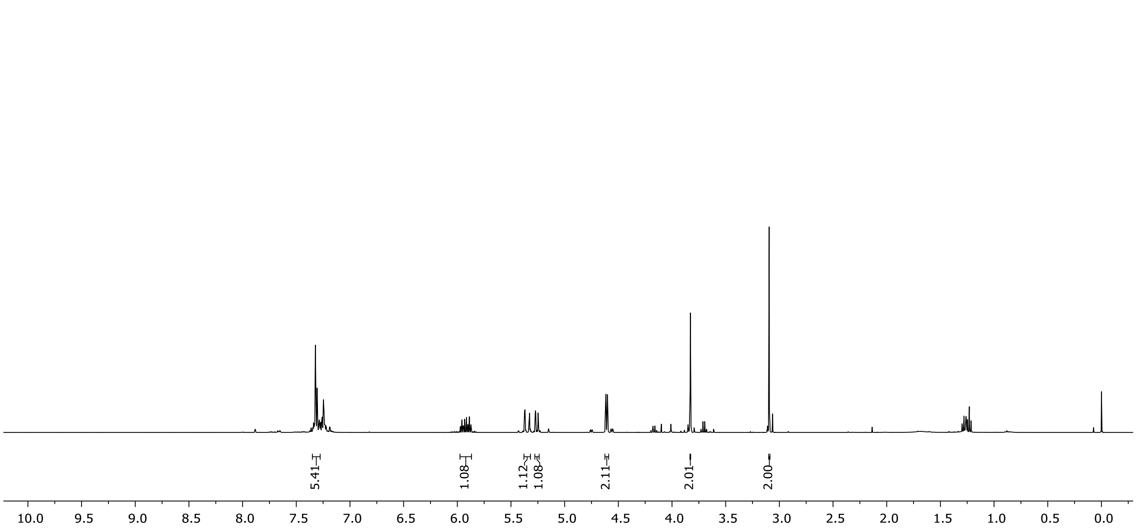

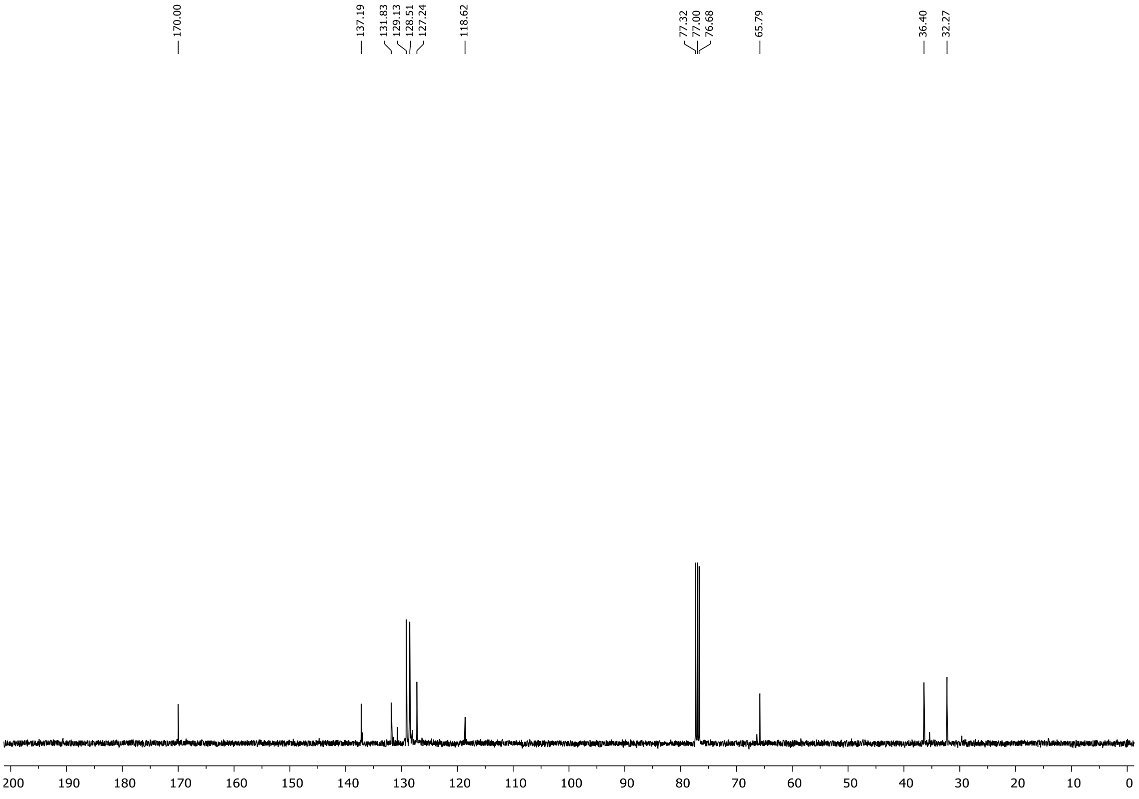

The ^{1}H (400 MHz) and ^{13}C (100 MHz) NMR spectra of **3q** in $CDCl_3$.

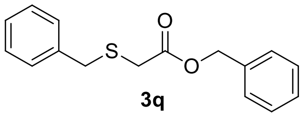

3q

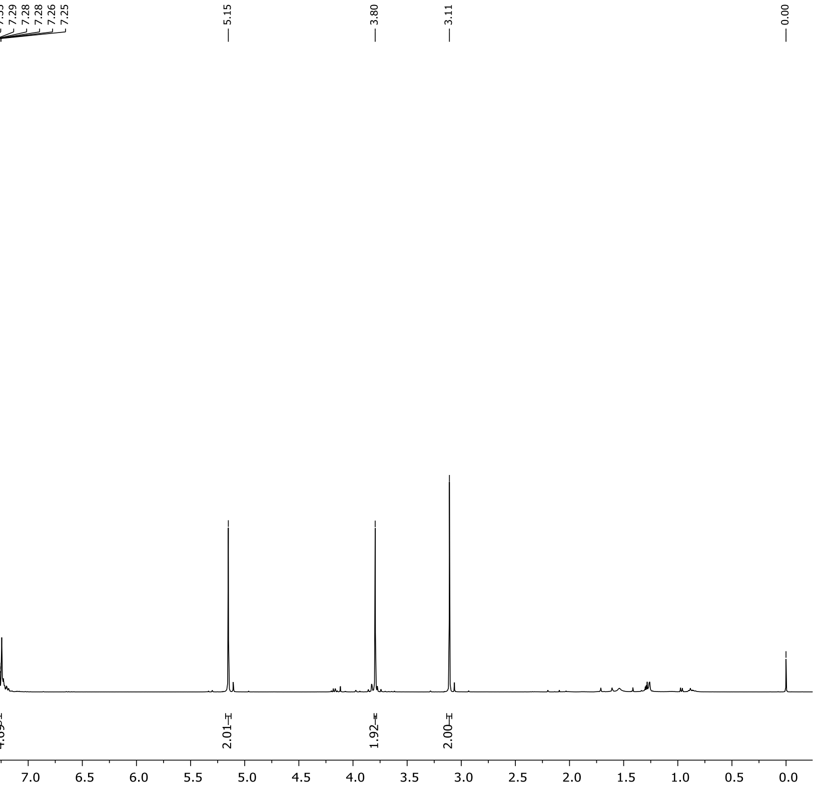

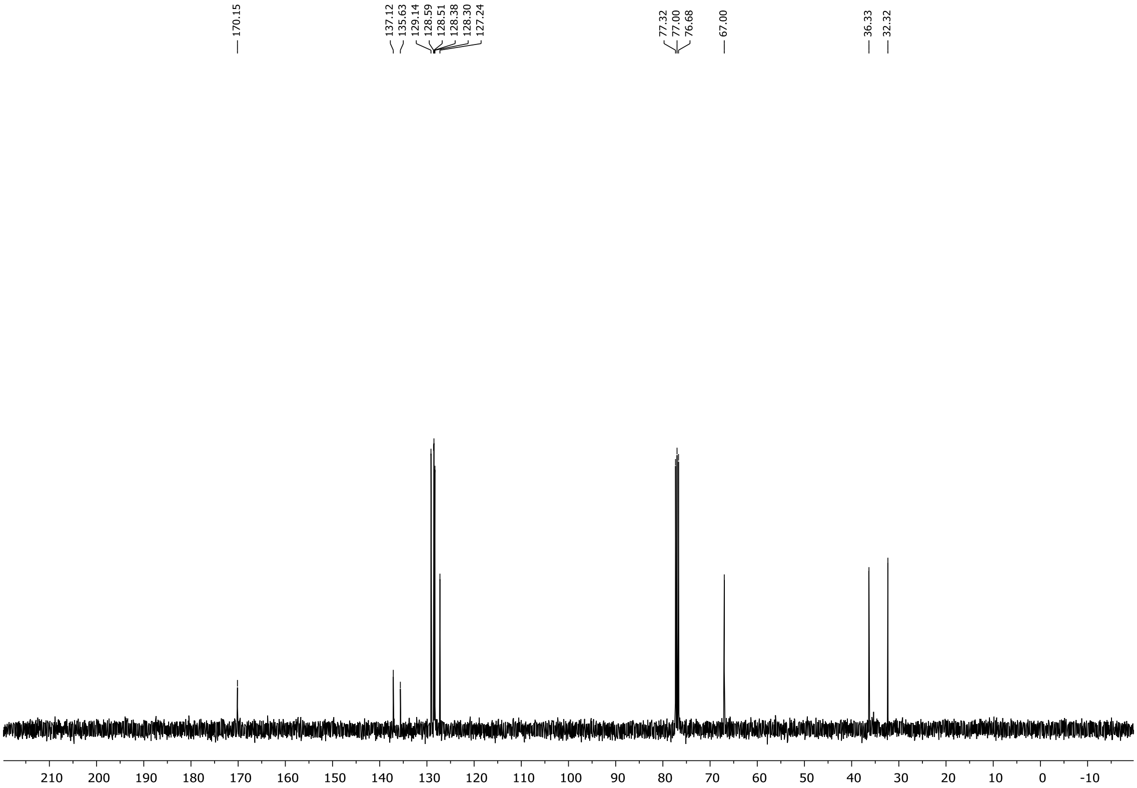

The ^{1}H (400 MHz) and ^{13}C (100 MHz) NMR spectra of **4a** in $CDCl_3$.

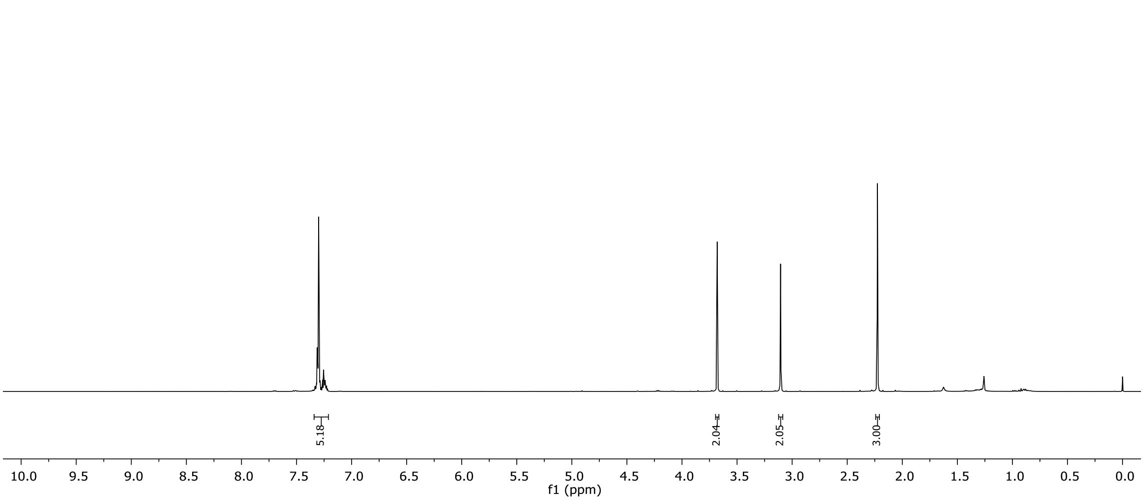

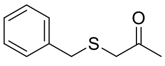

4a

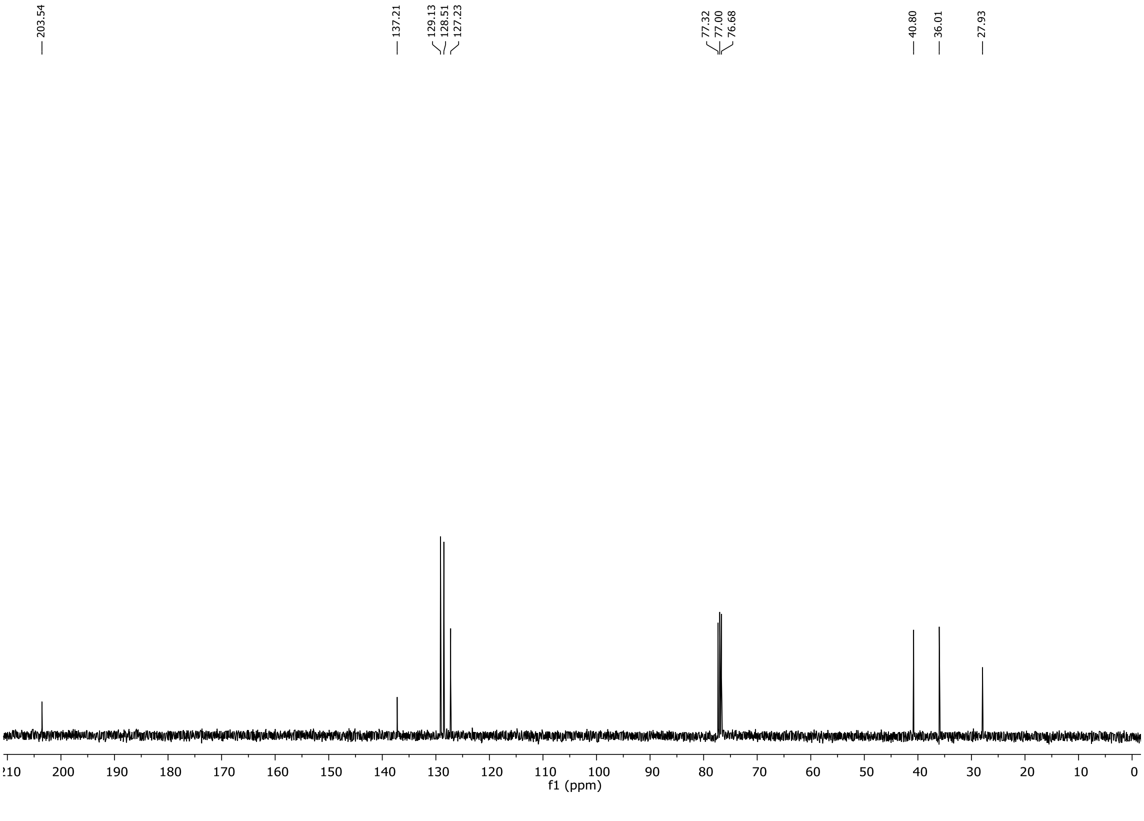

The ^{1}H (400 MHz) and ^{13}C (100 MHz) NMR spectra of **4b** in $CDCl_3$.

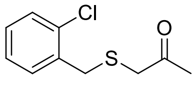

4b

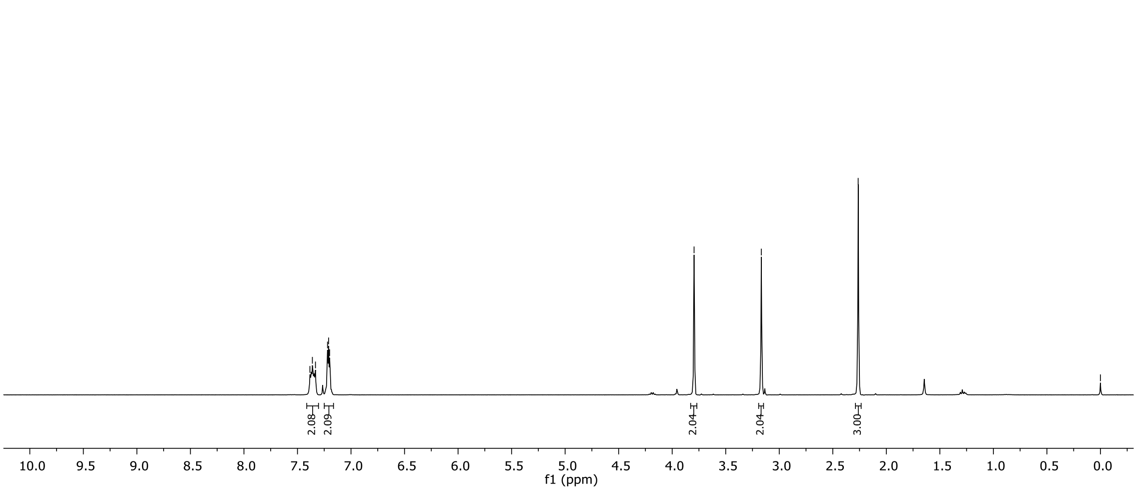

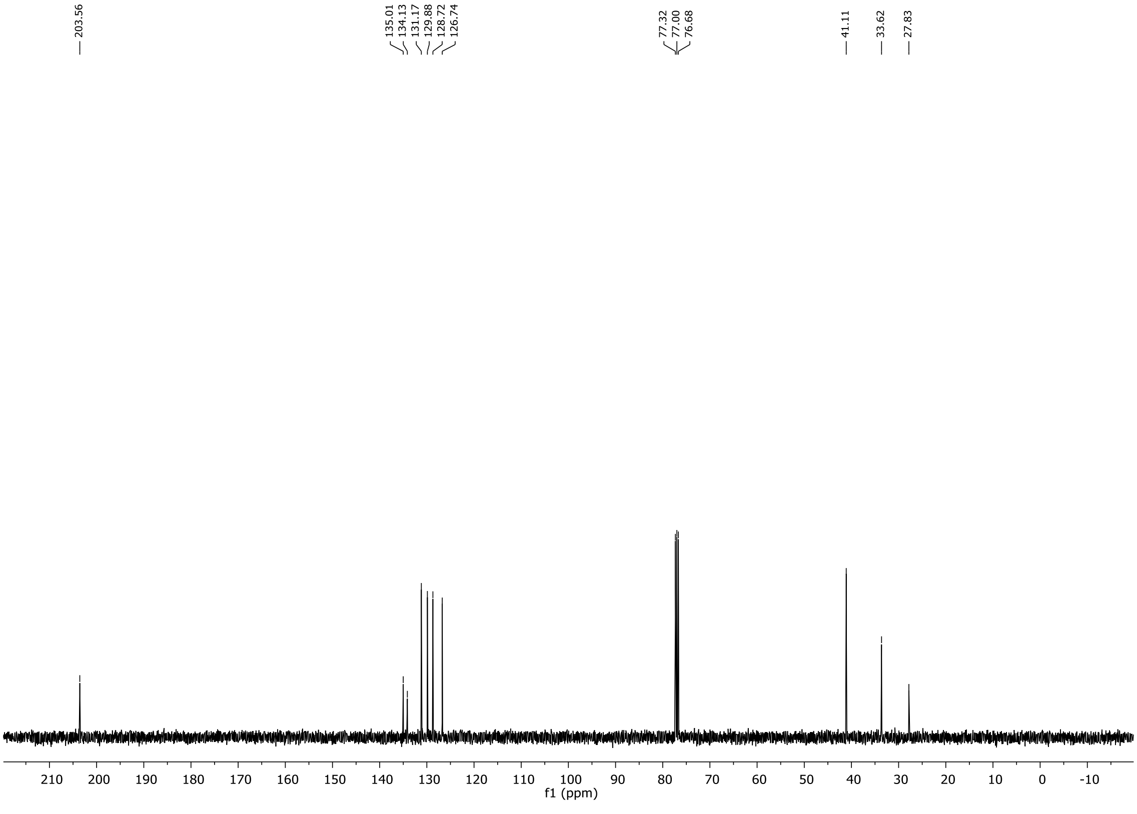

The ^{1}H (400 MHz) and ^{13}C (100 MHz) NMR spectra of **4c** in $CDCl_3$.

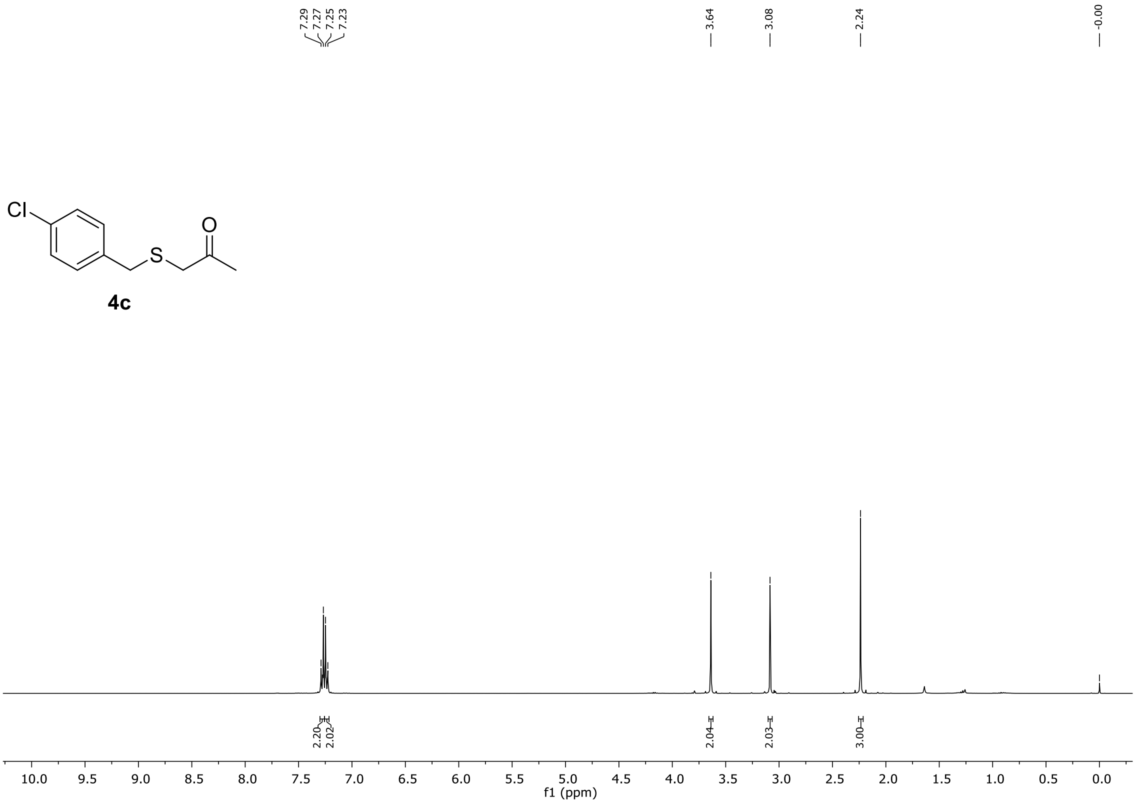

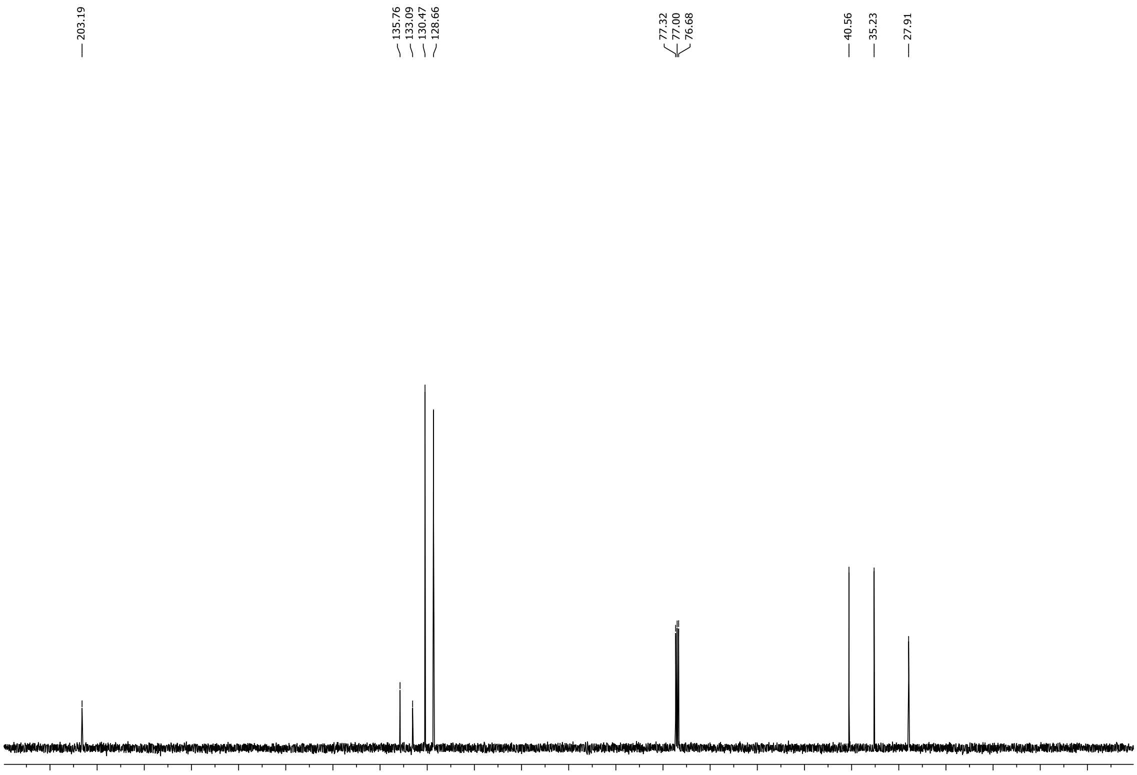

The ^{1}H (400 MHz) and ^{13}C (100 MHz) NMR spectra of **4d** in $CDCl_3$.

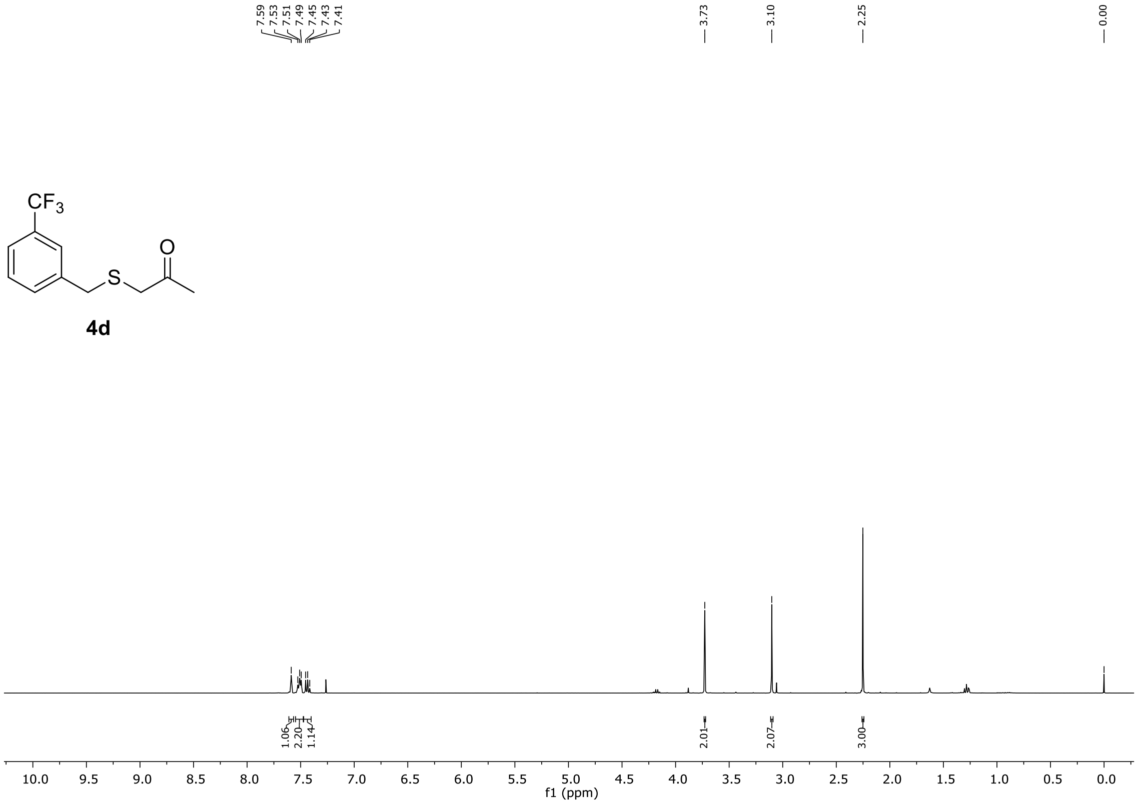

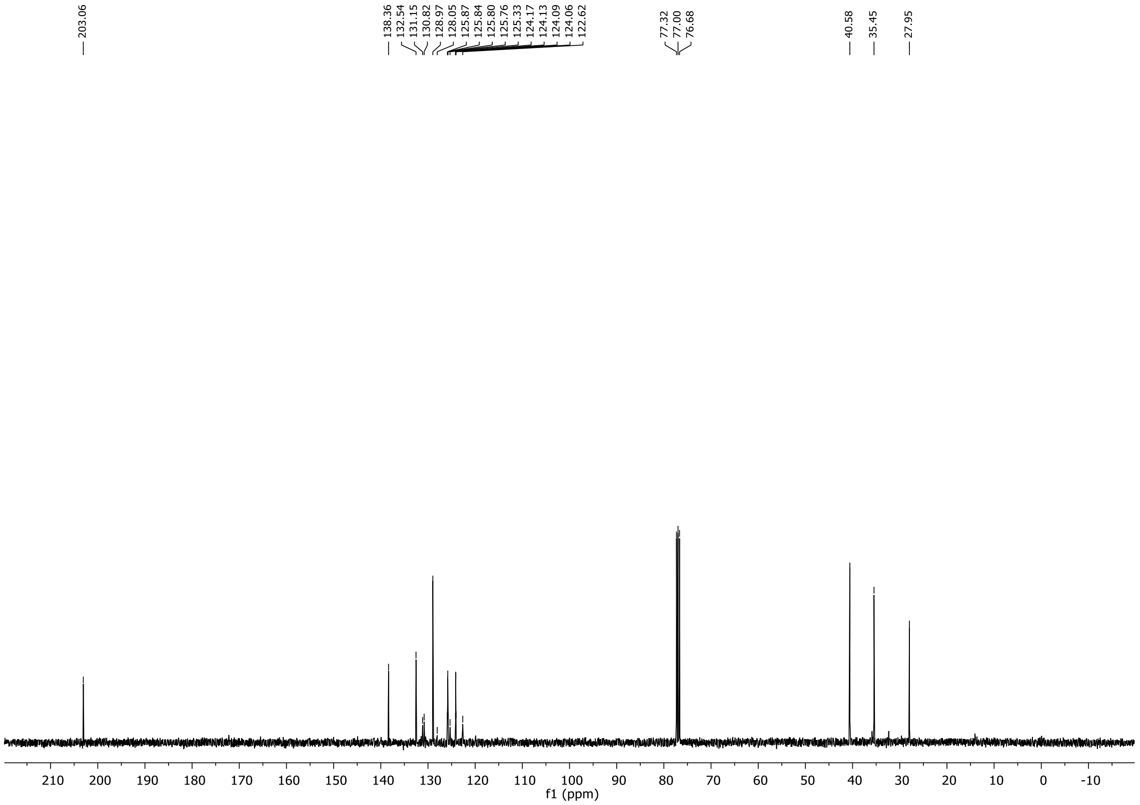

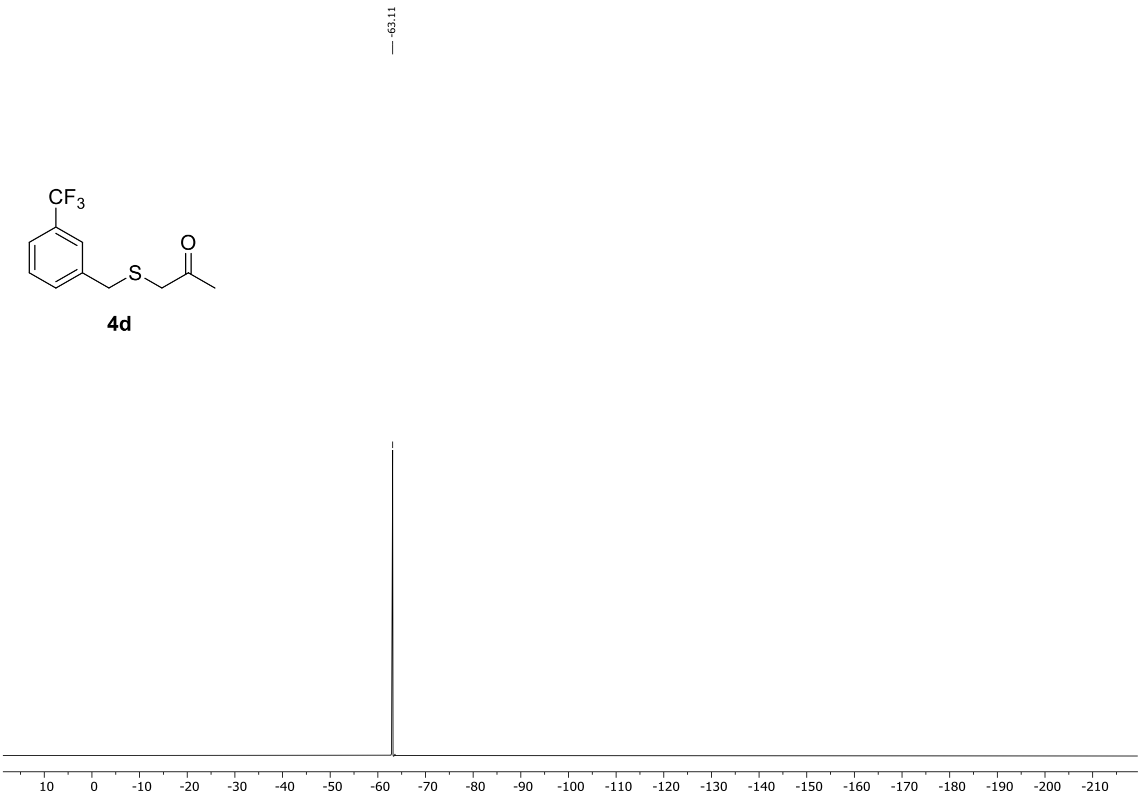
-63.11
CF3
S
O
10 0 -10 -20 -30 -40 -50 -60 -70 -80 -90 -100 -110 -120 -130 -140 -150 -160 -170 -180 -190 -200 -210

4d

The ^{1}H (400 MHz) and ^{13}C (100 MHz) NMR spectra of **4e** in $CDCl_3$.

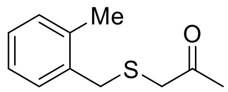

4e

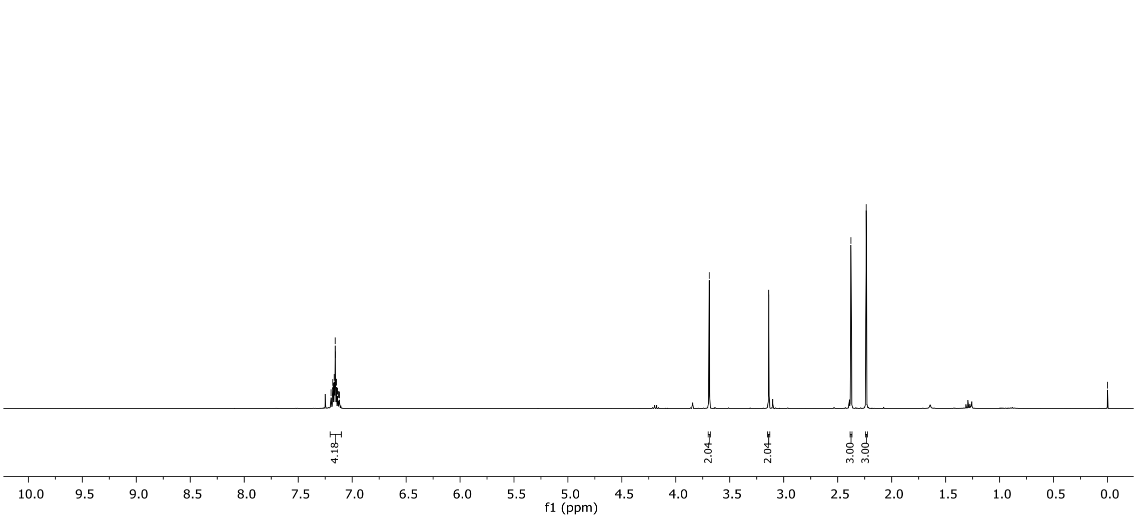

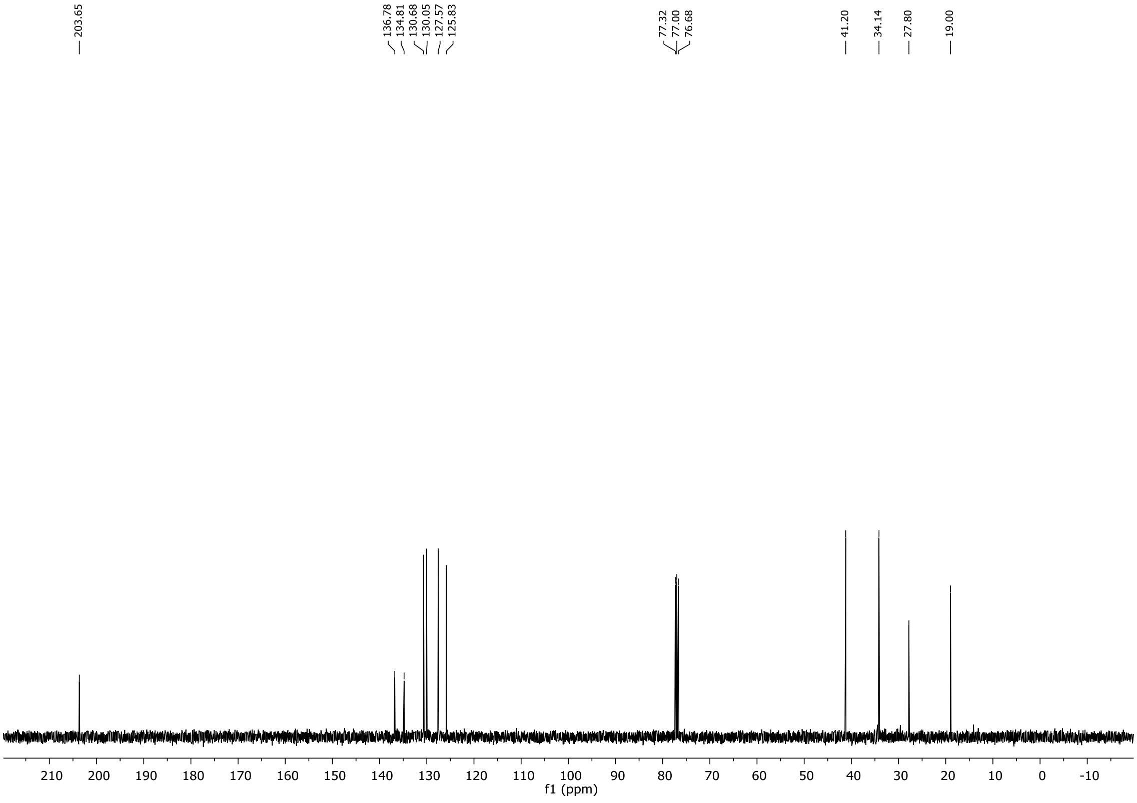

The ^{1}H (400 MHz) and ^{13}C (100 MHz) NMR spectra of **4g** in $CDCl_3$.

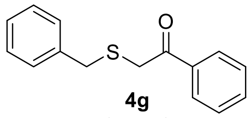

4g
(major)

+

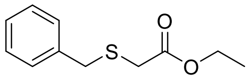

3a

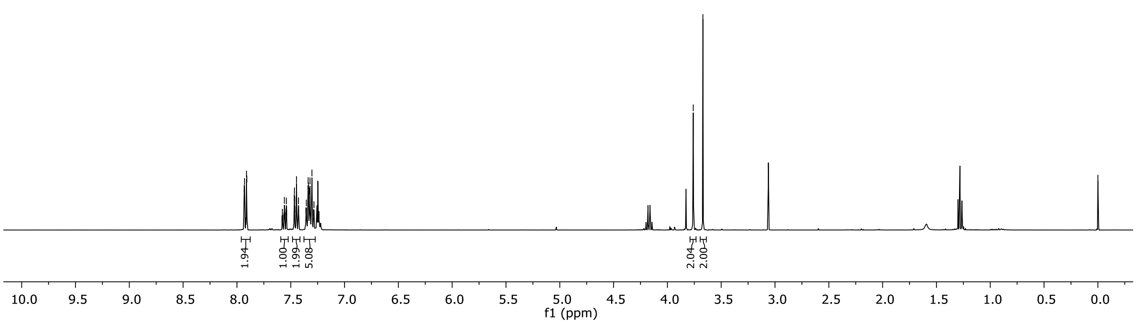

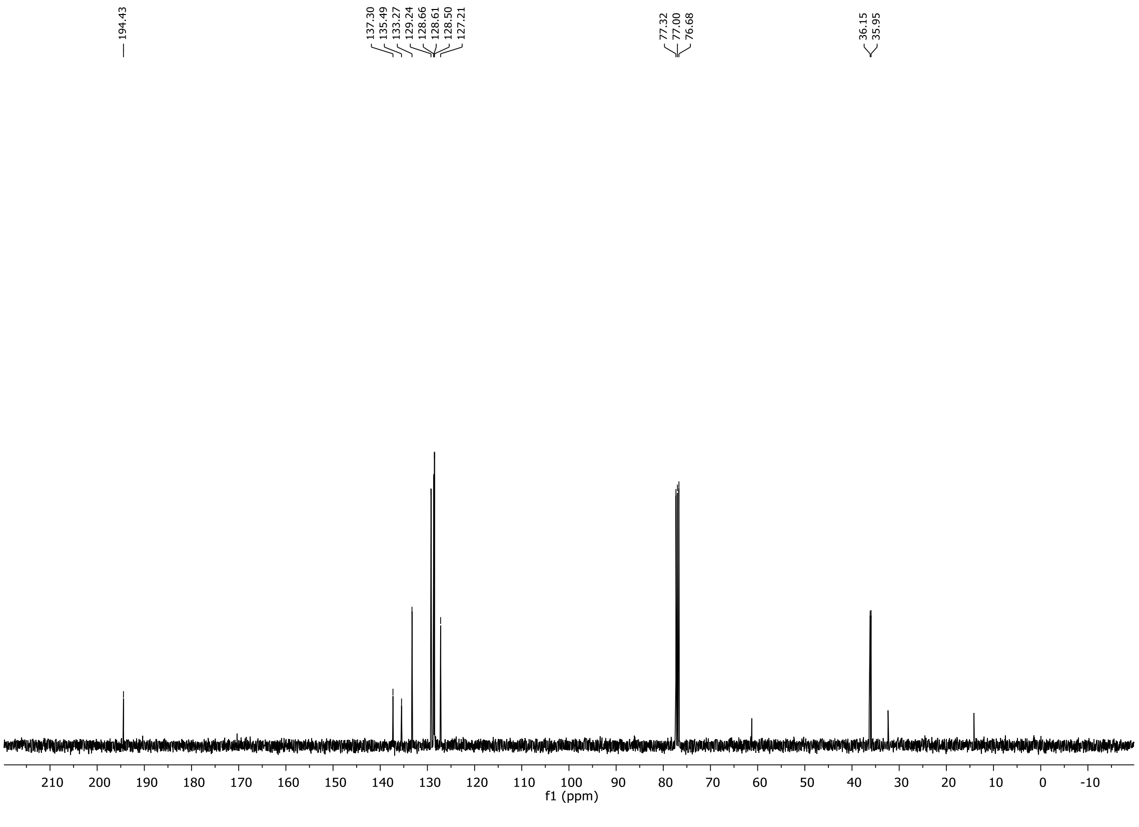

The ^{1}H (400 MHz) and ^{13}C (100 MHz) NMR spectra of **6** in $CDCl_3$.

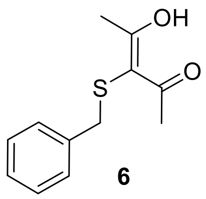

6

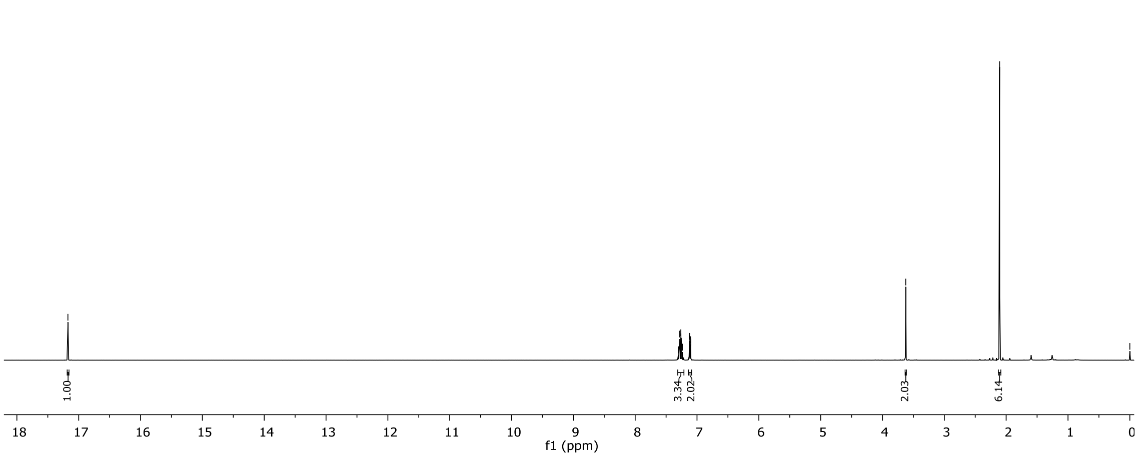

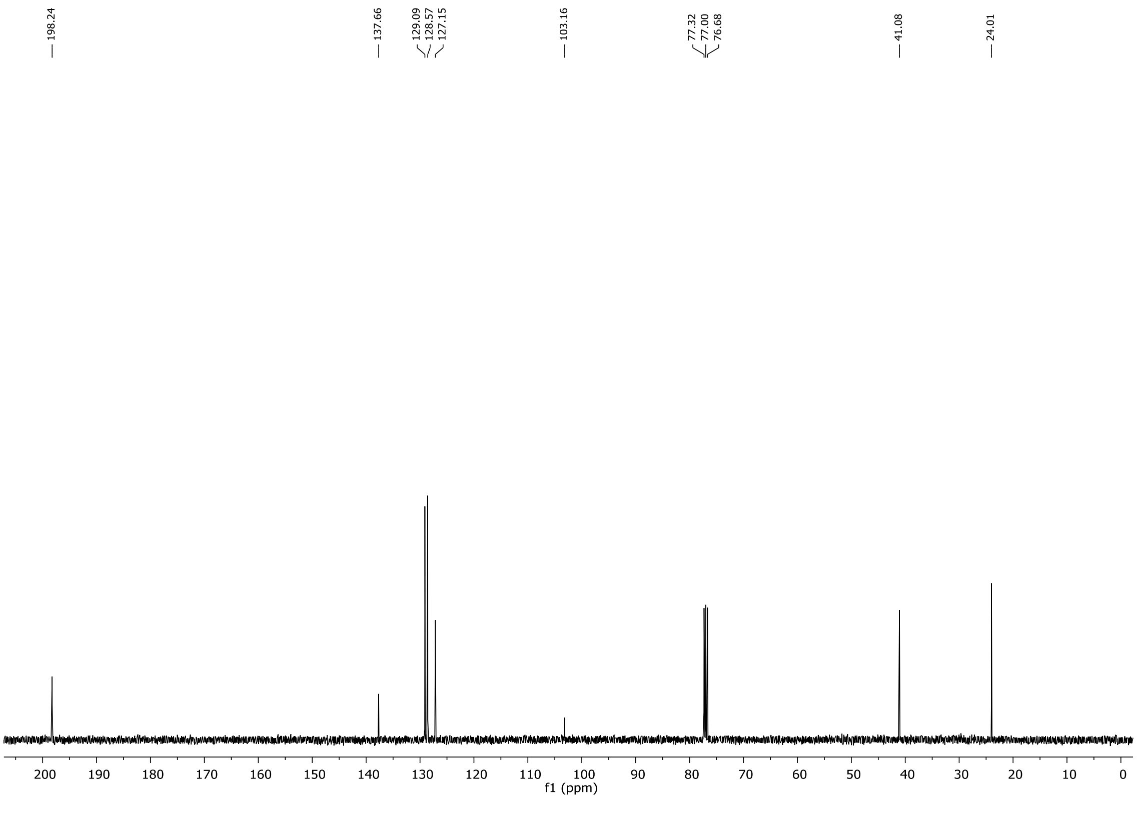

The ^{1}H (400 MHz) and ^{13}C (100 MHz) NMR spectra of **7** in $CDCl_3$.

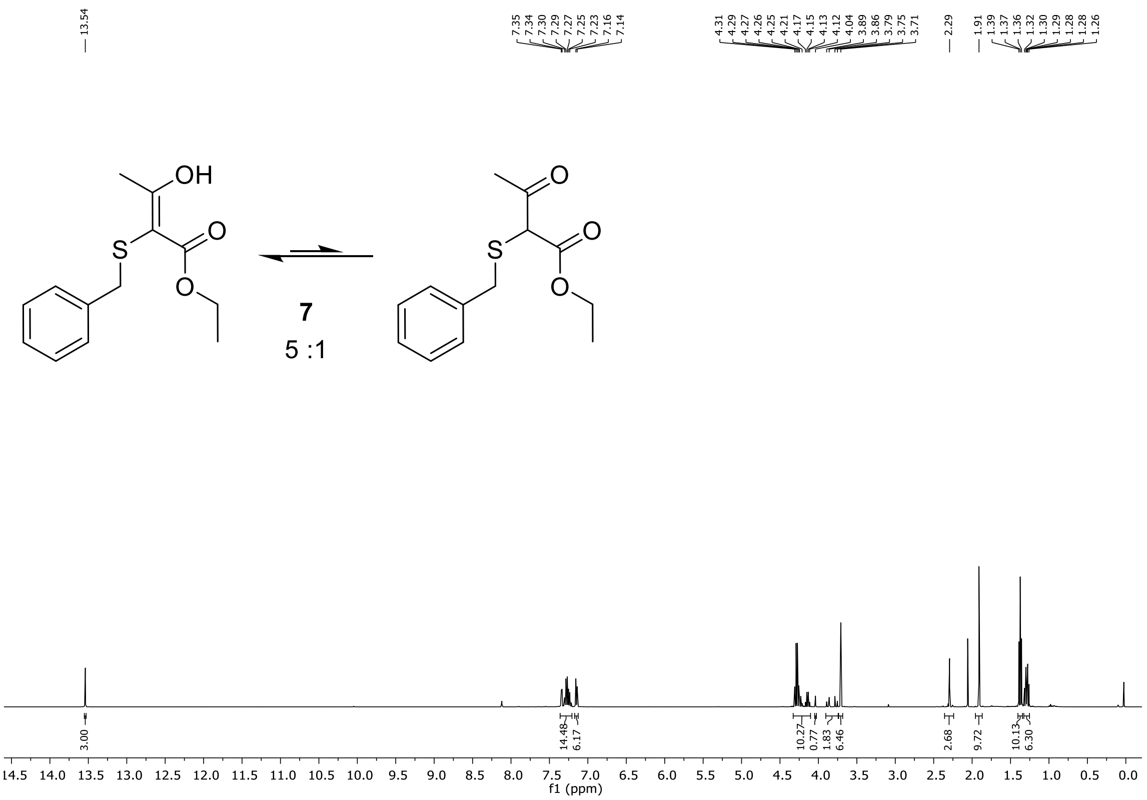

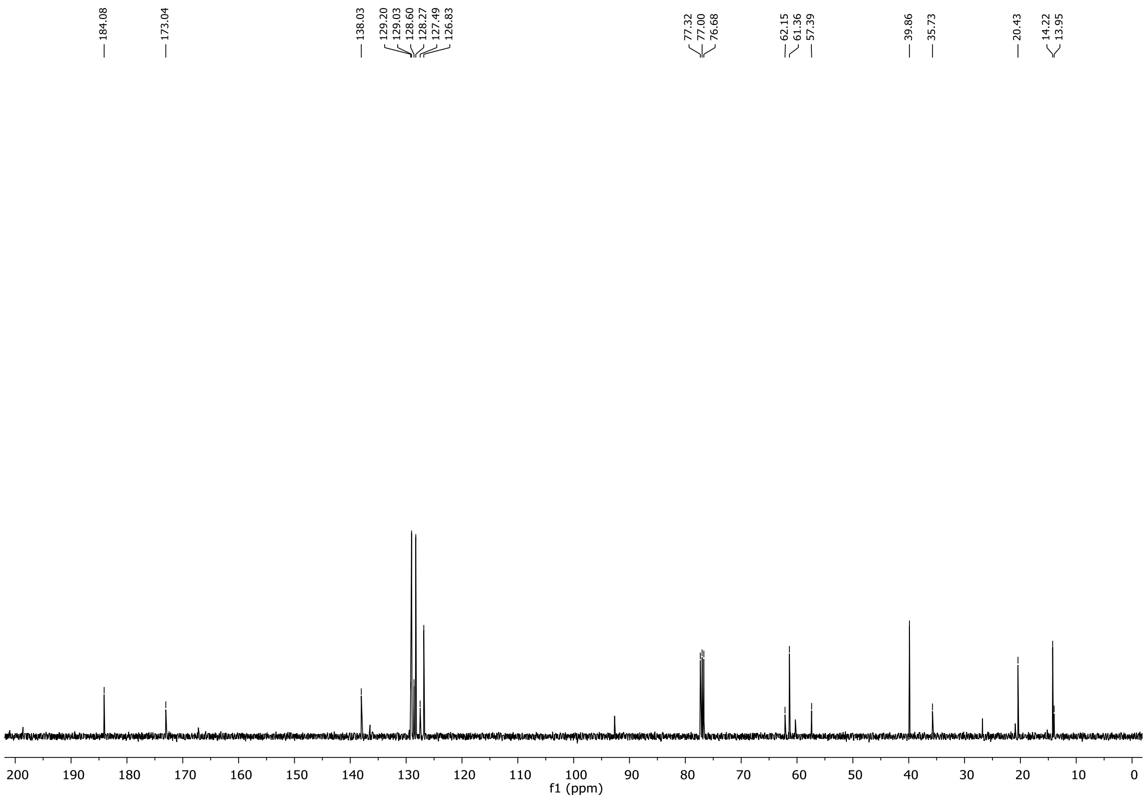

The ^1H (400 MHz) and ^{13}C (100 MHz) NMR spectra of **8** in $CDCl_3$.

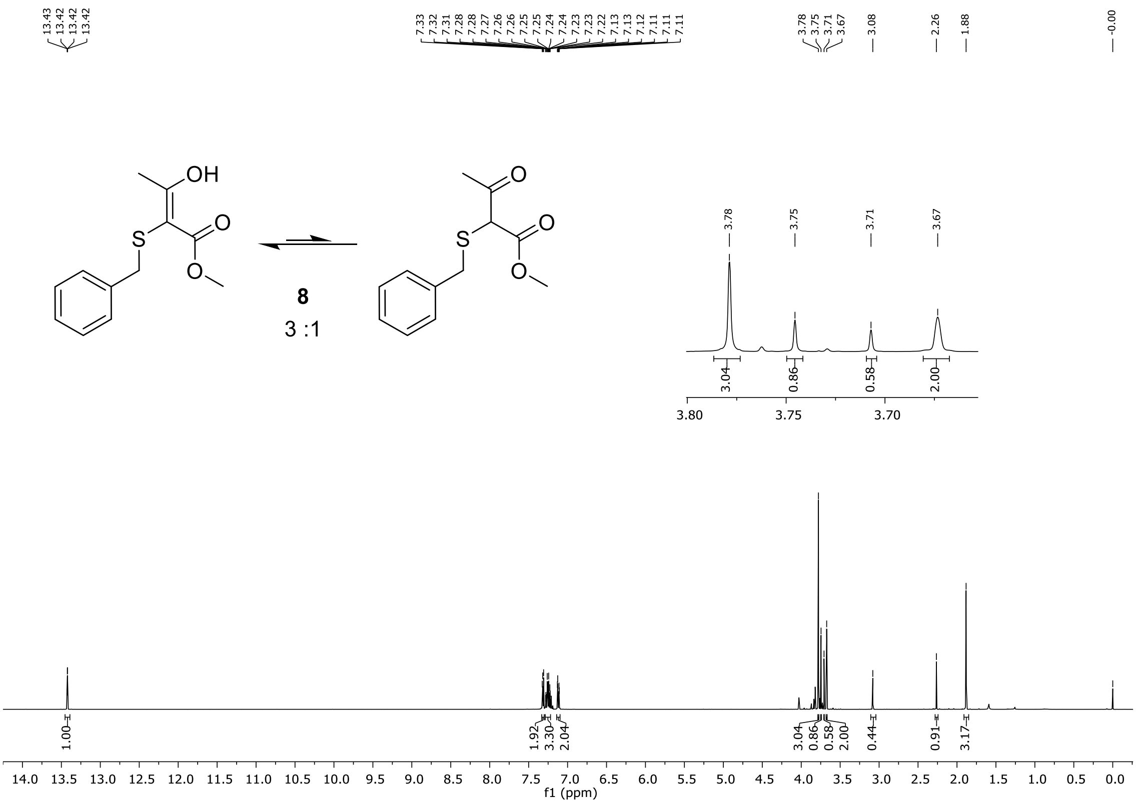

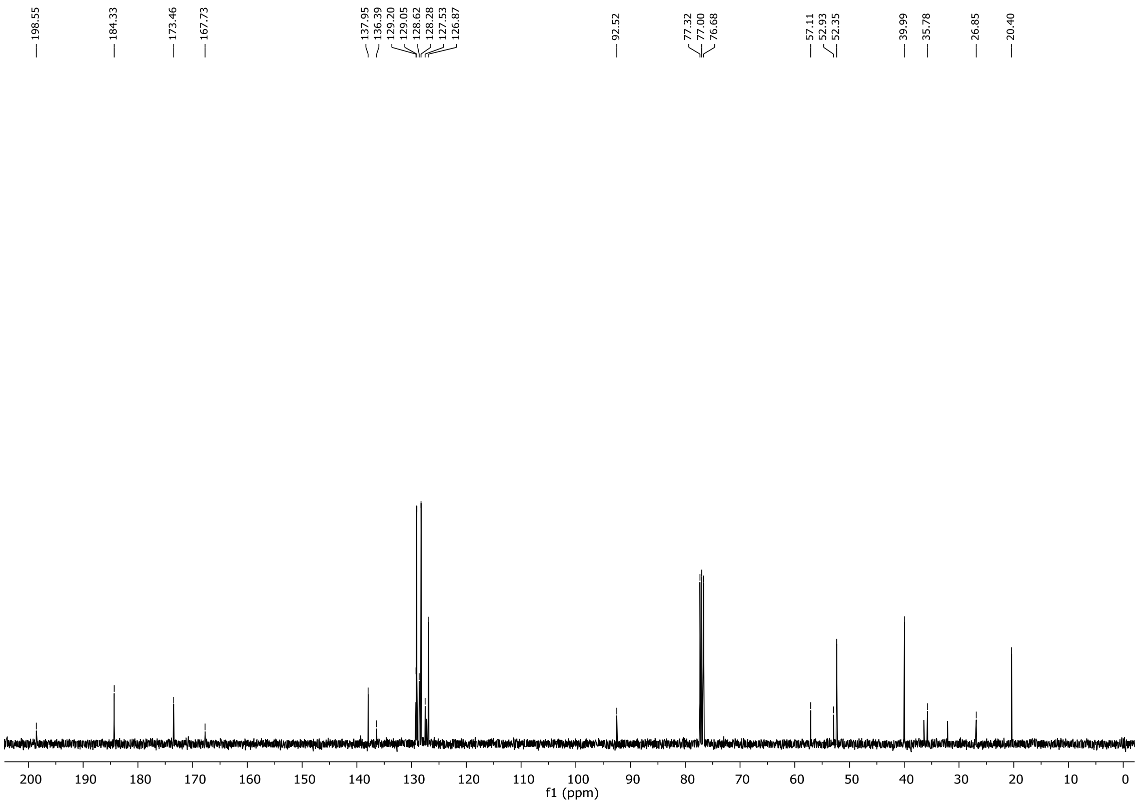

Supplement: File 1 — Experimental procedures, characterization data, control experiments, and copies of the 1H, 13C, and 19F NMR spectra. [file Beilstein_J_Org_Chem-17-234-s001.pdf]
